# Animal‐Free Setup of a 3D Mature Adipocyte‐Macrophage Co‐Culture to Induce Inflammation In Vitro

**DOI:** 10.1002/adhm.202500779

**Published:** 2025-06-20

**Authors:** Sophia Nowakowski, Svenja Nellinger, Franziska Brigitte Albrecht, Petra Juliane Kluger

**Affiliations:** ^1^ Reutlingen Research Institute Reutlingen University 72762 Reutlingen Germany; ^2^ Faculty of Natural Science University of Hohenheim 70599 Stuttgart Germany; ^3^ Faculty of Life Sciences Reutlingen University 72762 Reutlingen Germany; ^4^ Institute of Interfacial Process Engineering and Plasma Technology (IGVP) 70569 Stuttgart Germany; ^5^ Fraunhofer Institute for Interfacial Engineering and biotechnology IGB 70569 Stuttgart Germany

**Keywords:** adipose tissue disease models, defined culture media, gellan gum, immune cells

## Abstract

Adipose tissue inflammation plays a central role in the pathogenesis of metabolic disorders. It is closely associated with immune cell infiltration, particularly macrophages, and the release of pro‐inflammatory cytokines. Reliable in vitro test systems that mimic the inflamed environment while being free of animal‐derived components are essential to explore new treatments for obesity‐related diseases. This study aims to develop a straightforward, animal‐free adipocyte‐macrophage co‐culture for investigating adipose tissue inflammation. Therefore, the human monocytic cell lines Mono Mac (MM6) and THP‐1 are co‐cultured with human primary mature adipocytes (ACs) encapsulated in gellan gum (GG) within a defined environment. Both monocytic cell lines are effectively activated by phorbol 12‐myristate 13‐acetate (PMA) and lipopolysaccharide (LPS) in the defined medium, exhibiting distinct cytokine profiles. A comparison between collagen and GG demonstrates that GG is a suitable animal‐free matrix material for ACs. PMA+LPS successfully activates the 3D adipocyte‐macrophage co‐culture to an inflammatory state for 72 h in the developed defined medium. Viability and intracellular lipid content remain high, and the functionality of ACs (perilipin A) in untreated models remains intact. This inflamed adipocyte‐macrophage co‐culture is easy to assemble and set up in a defined environment, making it a potential test system for anti‐inflammatory treatment strategies.

## Graphical abstract

A completely animal‐free 3D co‐culture is developed using human fat cells and immune cells. Animal‐based materials are replaced with gellan gum hydrogel and a serum‐free medium. Immune cells are effectively activated, producing specific inflammatory signals. The co‐culture is easy to assemble, reproducible, and functions within 72 h, making it a promising tool for testing anti‐inflammatory therapies.

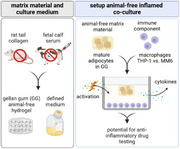

## 1 Introduction

Human adipose tissue contains ACs, adipose‐derived stem cells (ASCs), immune cells, and endothelial cells organized in lobules. Mature ACs regulate lipid metabolism by orchestrating triglyceride synthesis (lipogenesis) and breakdown (lipolysis).^[^ ^1^ ^]^ Previously viewed as a passive lipid reservoir, adipose tissue is now recognized as a dynamic organ influencing energy balance, systemic inflammation, and metabolism through the secretion of bioactive adipokines.^[^ ^2^ ^]^ Excessive fat accumulation results in an inflammatory environment characterized by the activation and infiltration of immune cells, including macrophages, antigen‐presenting dendritic cells, and T‐cells.^[^ ^3^ ^]^ Macrophages are recruited to dying adipocytes in a hypoxic environment due to the enlargement of adipocytes, leading to the release of inflammatory cytokines such as interleukin (IL)‐6, IL‐1β, and tumor necrosis factor α (TNFα).^[^ ^4^, ^5^ ^]^ T‐cells are activated by macrophages and dendritic cells, which contribute to a pro‐inflammatory state.^[^ ^6^ ^]^ In the lean state, adipose tissue macrophages display an anti‐inflammatory phenotype and support tissue homeostasis. During adipose tissue inflammation, adipose tissue macrophages significantly increase by up to 40–50% of the total cell count, and their polarization shifts to a pro‐inflammatory phenotype.^[^ ^7^, ^8^ ^]^ Incorporating the adipocyte‐macrophage crosstalk into in vitro models enhances their relevance, providing valuable insights into immune‐metabolic‐related diseases, like obesity, by mimicking the dynamic intercellular environment of adipose tissue.^[^ ^9^ ^]^

Various animal experiments, mainly in rodent models, have been conducted to study the prevention and therapy of inflammation‐related diseases.^[^ ^10^ ^]^ Despite the frequent use of animal models to study adipose tissue dysfunction, species variances in adipose tissue physiology must be considered, which could limit the transferability to humans.^[^ ^11^ ^]^ This highlights the increasing relevance of tissue‐engineered human in vitro test systems as alternatives for animal models in preclinical research.

**Figure 1:**
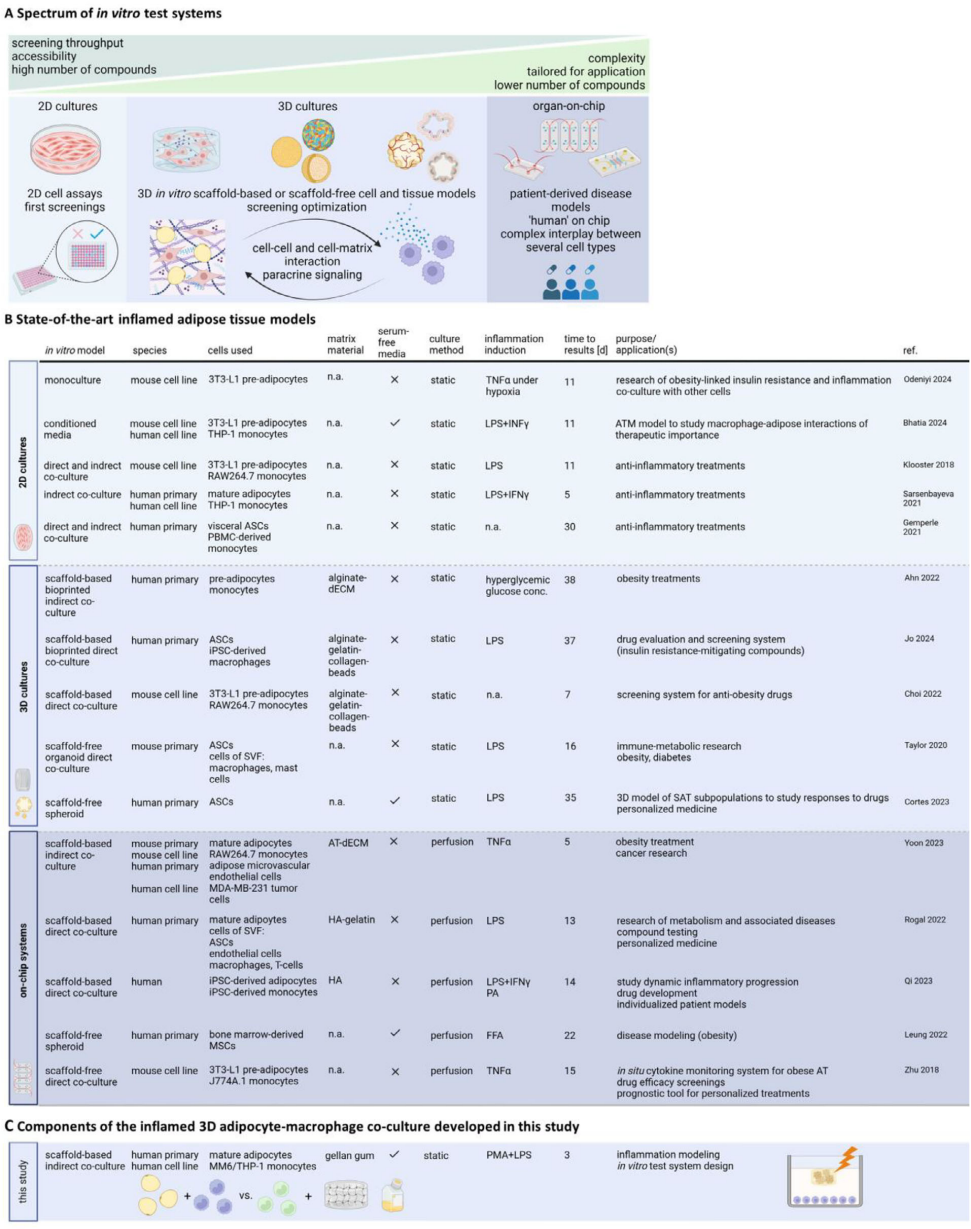
A) Schematic illustration of the spectrum of in vitro test systems for compound screening. B) Overview of state‐of‐the‐art inflamed adipose tissue models categorized by in vitro model structure, and C) positioning of the 3D adipocyte‐macrophage co‐culture developed in this study. SVF: stromal vascular fraction, HA: hyaluronic acid, dECM: decellularized extracellular matrix, AT: adipose tissue, iPSCs: induced pluripotent stem cells, d: days, ATM: adipose tissue macrophage, PBMC: peripheral blood mononuclear cells, MSCs: mesenchymal stem cells, n.a. not applicable, SAT: subcutaneous adipose tissue, IFNγ: interferon‐gamma.^[^ ^22^, ^34^, ^35^, ^36^, ^37^, ^38^ ^]^

Many configurations exist for modeling adipose tissue inflammation in vitro, and the model approach should be tailored to the research question addressed in terms of complexity and applicability (**Figure** **1**A,B). Physiological in vitro tissue models focus on replicating the full complexity of native tissues, providing insights into fundamental biological processes like cellular behavior. In contrast, test systems prioritize reproducibility and scalability, making them useful for substance screening and regulatory evaluations.^[^ ^12^ ^]^ These approaches differ in their focus on biological relevance versus practical applicability, and their integration enhances the predictive power of preclinical studies. Regarding the cells used for modeling adipose tissue in vitro, ACs could closely resemble native adipose tissue due to their fully differentiated status. In contrast, ASCs must be differentiated in vitro and, afterward, still exhibit an immature differentiation status compared to mature ACs, which might require more optimization of culture conditions.^[^ ^13^ ^]^ As an immune component for immune and metabolic regulation in adipose tissue, macrophages are an essential component of in vitro models aiming to replicate tissue functionality and disease states.^[^ ^14^ ^]^ These models rely on primary macrophages, directly isolated from tissues or blood, or macrophage cell lines derived from immortalized cells. While primary macrophages offer the advantage of closely mimicking the physiological phenotype and heterogeneity observed in vivo, their variability between donors and limited lifespan can complicate reproducibility and scalability. In contrast, macrophage cell lines provide a more consistent, reliable, and easily manageable cell source, enabling more standardized protocols.^[^ ^15^ ^]^ In in vitro models, inflammation is induced to create diseased characteristics through enhanced pro‐inflammatory signals with various activation stimuli reported. Inflammatory inducers include bacterial components, such as LPS or cytokines, which provoke an immune response in vivo and in vitro.^[^ ^16^ ^]^ Other stimulants related to obesity consist of an excess of free fatty acids, triggering inflammatory reactions.^[^ ^17^ ^]^

For 3D adipose tissue culture, different approaches like scaffold‐free in vitro models or scaffold‐embedded ASCs or ACs in hydrogels are applied.^[^ ^18^ ^]^ Hydrogels are 3D networks of different polymers with a high water‐binding capacity in their cross‐linked structure.^[^ ^19^ ^]^ They can resemble the mechanical properties of tissues and offer a tissue‐specific environment with adjustable stiffness and pore size while being ready‐to‐use and more easily accessible than the extracellular matrix (ECM).^[^ ^20^ ^]^ Naturally derived or synthetic xeno‐free hydrogels, including GG, alginate, hyaluronan, and polyethylene glycol, have emerged as promising alternatives to animal‐derived ECM components.^[^ ^21^, ^22^, ^23^, ^24^, ^25^ ^]^ GG was selected for the present study due to previous findings from our group, which demonstrated its suitability by generating GG‐based adipose‐like microtissues or a bioprinted setup of long‐term stable, functional 3D adipose tissue models.^[^ ^26^, ^27^ ^]^ GG is a bacteria‐derived exopolysaccharide and a promising material in tissue engineering due to its biocompatibility, non‐toxicity, and tunability.^[^ ^21^, ^28^ ^]^ Further, it is thermosensitive and cross‐linkable with mono‐ and divalent cations, which allows the physical properties of GG hydrogels to be modified by tailoring GG concentrations and cation content.^[^ ^29^, ^30^ ^]^

While GG hydrogels serve as an animal‐free matrix material for human adipose tissue models, fetal calf serum (FCS) and other animal‐derived components in cell culture media must also be critically evaluated. FCS is still considered the gold standard in cell culture media due to its complex composition of growth factors, hormones, and nutrients that support cell proliferation and viability. However, besides ethical issues, FCS can mask experimental outcomes in in vitro models and limit study comparability.^[^ ^31^, ^32^ ^]^ Moreover, animal‐derived components are prone to high batch‐to‐batch variations, which impair the reproducibility and reliability of such in vitro systems. However, most studies using completely defined media were solely for differentiation or the differentiation and maintenance of ASCs.^[^ ^33^ ^]^ Therefore, developing animal‐free and defined media for maintaining mature ACs is important to increase the validity of in vitro adipocyte‐macrophage co‐cultures.

In this work, we developed an easy‐to‐assemble and reproducible inflamed human mature adipocyte‐macrophage co‐culture using animal‐free matrix material and a defined medium developed in‐house (Figure 1C). Our approach includes ready‐to‐use primary mature ACs in GG hydrogels and compares two monocytic cell lines (MM6 and THP‐1) as immune components for short‐term results within 72 h. The induction of pro‐inflammatory processes in this in vitro test system makes it a suitable candidate for pharmacological studies related to anti‐inflammatory treatments.

## 2 Results

### 2.1 Monocytic Cell Lines MM6 and THP‐1 can be Cultured in the Defined Medium and Show Distinct Cytokine Profiles upon Activation

**Figure 2:**
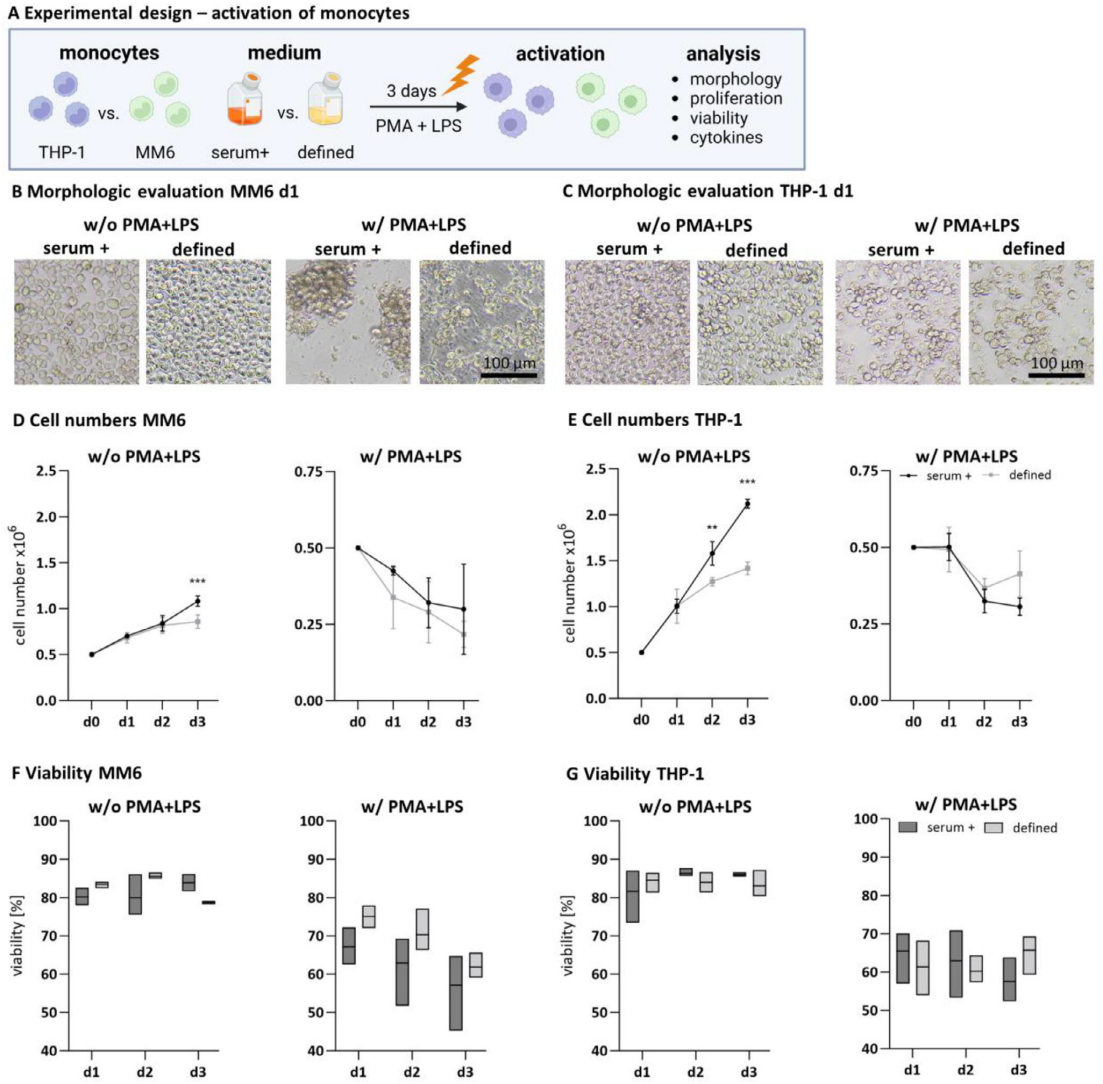
Monocyte (MM6 and THP‐1) activation in serum‐containing and defined medium in a monoculture over 3 days. A) Schematic illustration of experimental design testing the monocytic cell lines MM6 and THP‐1 in serum‐containing and defined medium with activating stimuli PMA and LPS. B) Phase contrast images of MM6 and C) THP‐1 activated by PMA+LPS in the serum‐containing medium (serum +) and the defined medium (defined) after 24 h. Scale bar 100 µm. D) Cell numbers of MM6 and E) THP‐1 over 3 days in the respective medium. Cell numbers are displayed as x10^6^ cells. F) Viability [%] of unstimulated (w/o PMA+LPS) and stimulated (w/ PMA+LPS) MM6 or G) THP‐1 over 3 days in the respective medium. Mean values represent the mean ± standard deviation (SD) of three independent experiments with distinct cell batches. ^**^ *p* ≤ 0.01, ^***^ *p* ≤ 0.001.

To test whether the activation of monocytes is possible with PMA and LPS for the defined inflamed co‐culture model, monocytes were stimulated with a combination of PMA and LPS as effective activators (**Figure** **2**A). Unstimulated monocytes served as the negative control. The clumping assay images from day 1 after stimulation show characteristic aggregation of MM6 in the PMA+LPS condition in both media (Figure 2B). It can be observed that the aggregates of MM6 were more distinct in the serum‐containing medium than in the defined medium. THP‐1 exhibited partially adhered cells and clumping in both media (Figure 2C). Regarding cell proliferation, unstimulated MM6 numbers were significantly higher in the serum‐containing (1.08 × 10^6^ ± 0.16) than in the defined medium (0.86 × 10^6^ ± 0.11) on day 3 (Figure 2D). Unstimulated THP‐1 showed lower cell numbers in the defined medium (1.42 × 10^6^ ± 0.16) compared to the serum‐containing medium (2.12 × 10^6^ ± 0.22) (Figure 2E). For both cell lines and in both media, PMA+LPS‐stimulation showed significantly lower cell numbers on all days compared to the unstimulated control. These results were accompanied by the viability analysis. Overall, viability was significantly reduced in the PMA+LPS‐stimulated condition compared to the unstimulated control for both cell lines and in both culture media (Figure 2F,G). For MM6 and THP‐1, the viability of the unstimulated control was similar in the serum‐containing (MM6: 83.89% ± 10.19, THP‐1: 85.99% ± 3.37) and defined medium (MM6: 78.71% ± 7.47, THP‐1: 83.06% ± 3.70) on day 3. PMA+LPS‐stimulated cells displayed reduced viability in both media, with 57.09% (± 14.31) for MM6, 57.56% (± 6.76) for THP‐1 in serum‐containing medium, and 58.40% (± 9.86) for MM6, 65.71% (± 8.59) for THP‐1 in the defined medium. PMA or LPS stimulation only reduced cell numbers for cell culture media and cell lines, whereas LPS had less cell number and viability‐reducing effect in THP‐1 (Figure S1, Supporting Information).

**Figure 3:**
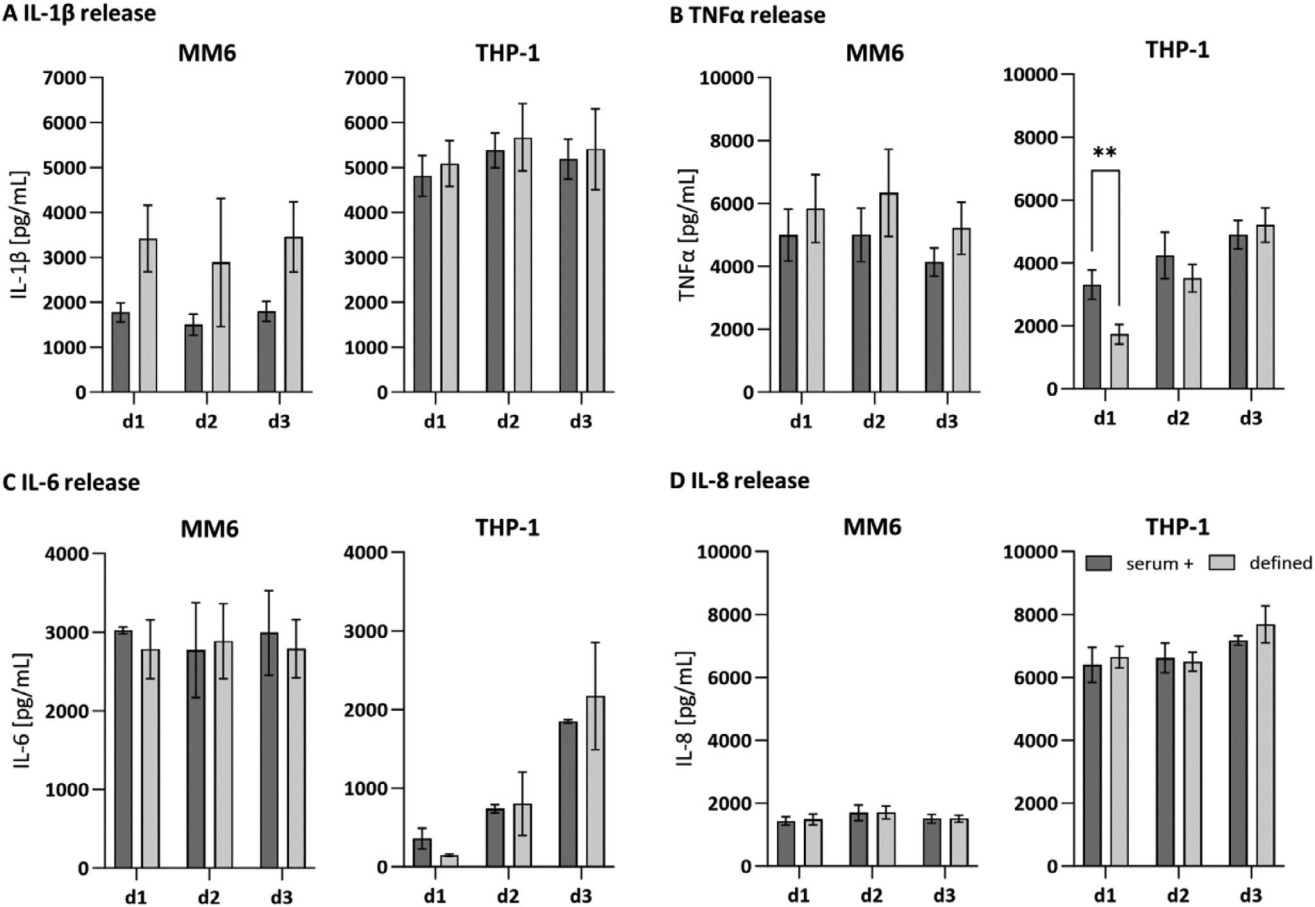
Release of cytokines A) IL‐1β, B) TNFα, C) IL‐6, and D) IL‐8 of PMA+LPS‐activated MM6 and THP‐1 in serum‐containing and defined medium over 3 days. Mean values represent the mean ± SD in pg mL^−1^ of three independent experiments. ^**^ *p* ≤ 0.01.

Inflammatory cytokine concentration of IL‐1β, TNFα, IL‐6, and IL‐8 was determined in the cell culture supernatants of MM6 and THP‐1 over 3 days post‐stimulation to quantify the activation quality. For MM6, IL‐1β levels differed between the serum‐containing medium (day 3: 1799.53 pg mL^−1^ ± 456.42) and the defined medium (day 3: 3451.65 pg mL^−1^ ± 1350.67), with higher concentrations in the defined medium and higher standard deviations (**Figure** **3**A **MM6)**. THP‐1 showed comparable concentrations of released IL‐1β over 3 days in the serum‐containing (5184.76 pg mL^−1^ ± 425.99) and the defined medium (5403.96 pg mL^−1^ ± 928.60), and exhibited higher overall values than MM6 (Figure 3A **THP‐1)**. TNFα secretion in MM6 was not significantly altered in the defined medium (5213.82 pg mL^−1^ ± 1043.64) compared to the serum‐containing medium (4134.69 pg mL^−1^ ± 668.40), with only a slight decrease on day 3. In stimulated THP‐1 cells, TNFα levels exhibited an increasing pattern over 3 days in both culture media with higher starting levels in serum‐containing medium on days 1 and 2 (serum day 1: 3312.47 pg mL^−1^ ± 931.51, defined day 1: 1734.38 pg mL^−1^ ± 373.53) and similar levels on day 3 compared to the defined medium (serum day 3: 4901.52 pg mL^−1^ ± 507.21, defined day 3: 5204.89 pg mL^−1^ ± 706.31) (Figure 3B). IL‐6 concentrations in stimulated MM6 were nearly constant over 3 days in both culture media (serum day 3: 2991.22 pg mL^−1^ ± 648.48, defined day 3: 279 pg mL^−1^ ± 416.79). THP‐1 cells exhibited a delayed increase upon the 3‐day analysis period, with the highest levels on day 3 in both culture media (serum + day 3: 1847.61 pg mL^−1^ ± 163.70, defined day 3: 2172.04 pg mL^−1^ ± 596.60) and higher standard deviations in the defined medium (Figure 3C). For IL‐1β, TNFα, and IL‐6, levels of the unstimulated control were below the detection limit (unstimulated levels of IL‐8 in supplements). MM6 cells released nearly constant levels of IL‐8 over the 3 days in both culture media (serum day 3: 1510.09 pg mL^−1^ ± 172.86, defined day 3: 1509.49 pg mL^−1^ ± 142.20), with detectable levels also for the unstimulated control (serum day 3: 484.88 pg mL^−1^ ± 87.81, defined day 3: 341.71 pg mL^−1^ ± 41.45). Detected IL‐8 levels for THP‐1 were nearly over 3‐fold higher than for MM6 in both culture media (serum day 3: 7173.15 pg mL^−1^ ± 607.17, defined day 3:7684.32 pg mL^−1^ ± 614.41) (Figure 3D). Basal release of IL‐8 for THP‐1 was only detected in the defined medium (defined day 3: 455.15 pg mL^−1^ ± 176). PMA or LPS stimulation only showed varying activation capacity in MM6 and THP‐1 for the different cytokines (Figure S2, Supporting Information). The clumping assay, viability analyses, and the quantification of the inflammatory cytokines after PMA+LPS‐stimulation of MM6 and THP‐1 showed acceptable viability and indicated a successful activation. The activation levels were mainly comparable between the serum‐containing and the defined medium but differed between the cell lines MM6 and THP‐1.

### 2.2 Gellan Gum is a Suitable Animal‐Free Biomaterial for the Defined Culture of Encapsulated Adipocytes

**Figure 4:**
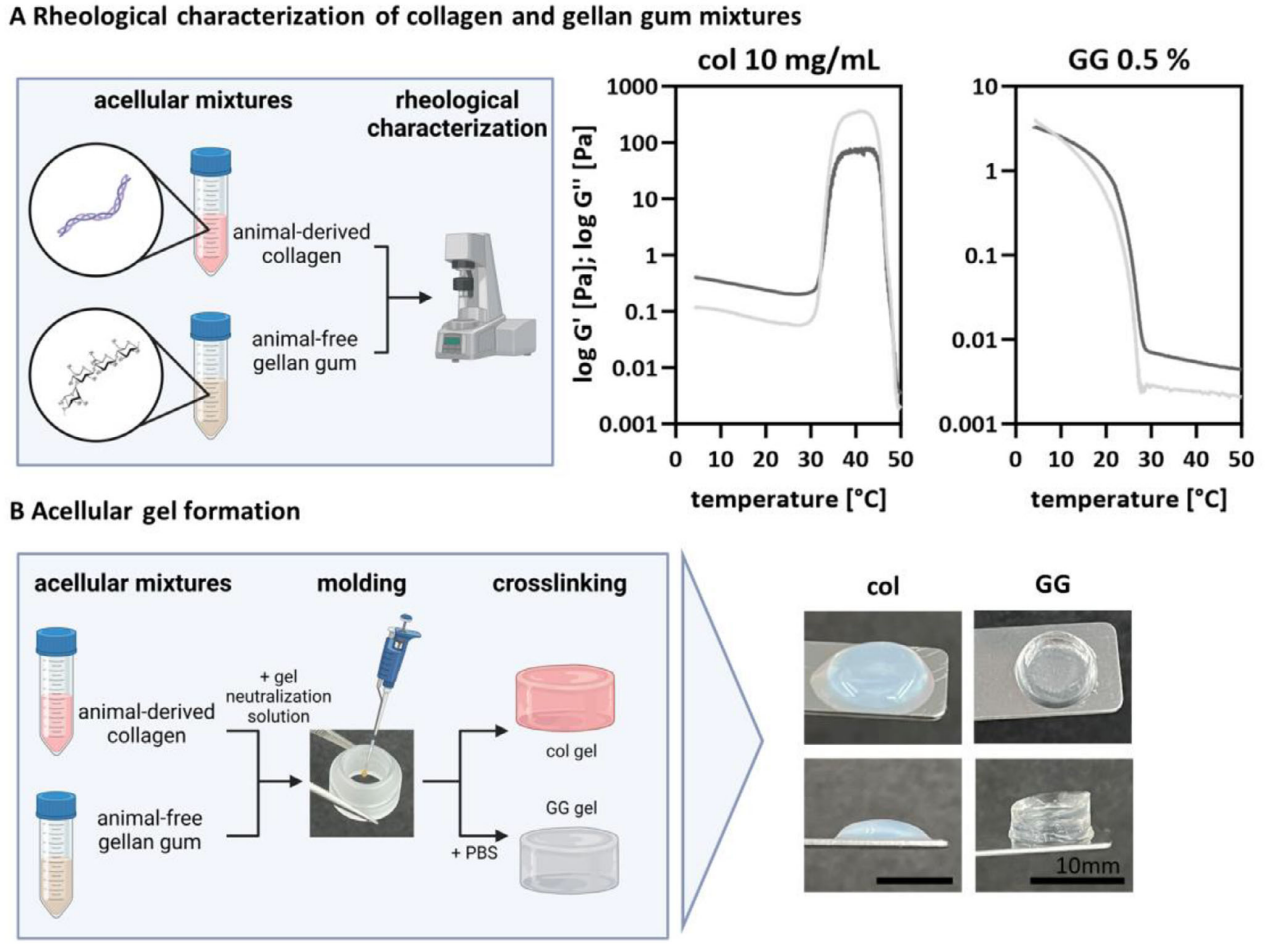
Rheological characterization of acellular collagen and gellan gum mixtures as matrix materials for AC encapsulation. A) Schematic illustration of experimental design of rheological measurements of col and GG mixtures. Oscillatory rheological measurements of acellular col (10 mg mL^−1^) and GG (0.5%) mixtures at different temperatures. B) Schematic illustration of acellular gel formation. Macroscopic pictures of acellular cross‐linked col and GG hydrogels. Scale bar 10 mm. col = collagen, GG = gellan gum.

In adipose tissue engineering, collagen of various species is widely used as a scaffold material. However, it is an animal‐derived and undefined matrix material to set up a defined adipose tissue model. Therefore, a non‐animal‐derived matrix material that maintains stability and cell functionality is needed. As the stiffness of the matrix affects AC stability, different concentrations of GG were tested for encapsulation of ACs (Figure S3, Supporting Information).^[^ ^39^ ^]^ A starting concentration of 0.5% GG showed the best processing and lipid vacuole integrity results and was therefore selected for further experiments. Non‐crosslinked acellular collagen and GG mixtures were characterized by rheological measurements (**Figure** **4**A). The sol–gel transition of the collagen and GG pre‐solution was determined with an oscillatory rheological measurement. The collagen's gelation point (crossing point of G’ and G’’) is at 32 °C and that of GG at 8 °C. While the elastic and viscous modulus decrease with increasing temperature for GG, both moduli are relatively constant until the sol–gel transition for collagen. After this point, both moduli increase until 47 °C and quickly decrease afterward. The mechanical properties, determined via the complex shear moduli G^*^, have a value of 57.35 Pa for collagen and 2.03 Pa for GG. Macroscopic pictures of the acellular collagen and GG hydrogels revealed differences in gel appearance and behavior. Collagen‐based gels were turbid and less dimensionally stable than the completely clear and non‐shrinkable GG gels with the same volume (Figure 4B).

**Figure 5:**
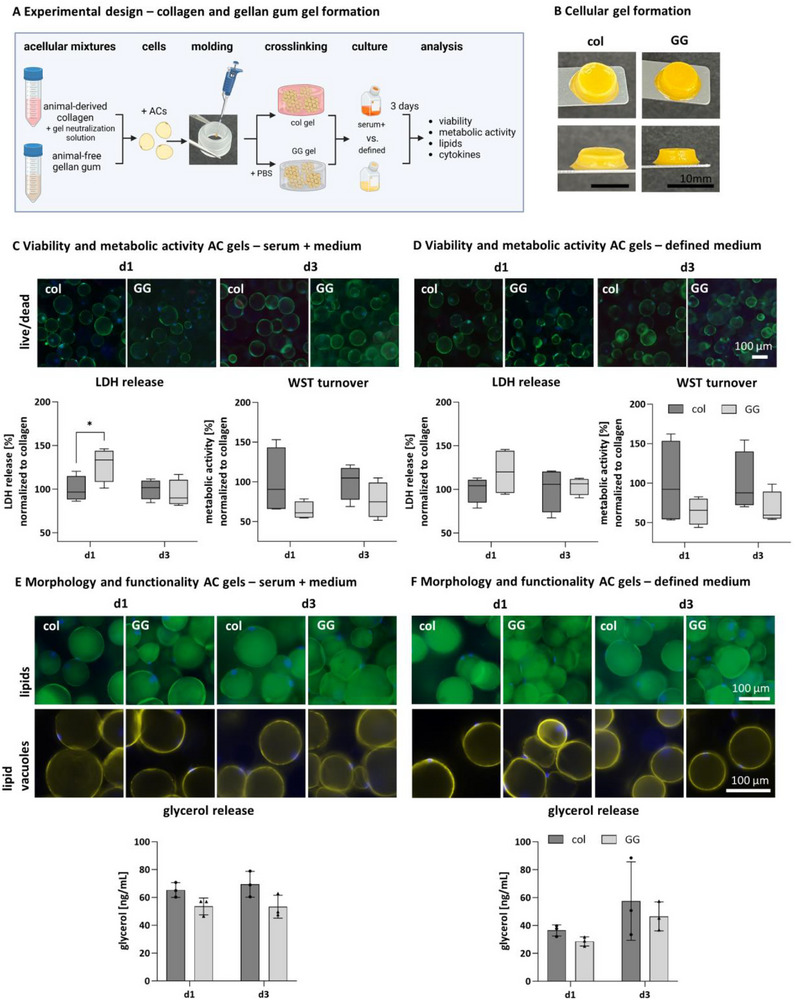
Comparison of collagen and gellan gum for encapsulation of mature adipocytes in serum‐containing and defined medium on days 1 and 3. A) Schematic illustration of experimental design for the formation of cross‐linked cell‐laden hydrogels. B) Macroscopic pictures of AC‐containing hydrogels. Scale bar 10 mm. C) Live/dead staining of AC‐col and AC‐GG hydrogels in serum‐containing medium and D) the defined medium, living cells in green, dead cells in red, and nuclei in blue, scale bar 100 µm. Normalized LDH release of encapsulated ACs in col and GG hydrogels. Normalized metabolic activity (by WST assay) of AC‐col and AC‐GG hydrogels (collagen set to 100%). E) Visualization of intracellular lipids and lipid vacuoles of encapsulated ACs in col and GG hydrogels at days 1 and 3. Lipids stained with BODIPY, lipids in green, nuclei in blue, lipid vacuoles stained for perilipin A, lipid vacuoles in yellow, nuclei in blue. Scale bar 100 µm. Basal glycerol release of ACs in collagen and GG hydrogels (in ng mL^−1^) in serum‐containing and F) the defined medium. col = collagen, GG = gellan gum. Serum + = serum‐containing medium, defined = defined medium. Mean values represent the mean ± SD of three independent experiments with three different biological donors. ^*^ *p* ≤ 0.05.

The viability and metabolic activity of ACs encapsulated in GG were analyzed on days 1 and 3 and compared to ACs encapsulated in collagen type I. Both models were cultured in serum‐containing adipocyte medium and the defined medium. To demonstrate that GG is a suitable material for maintaining the functionality of mature ACs comparable to the standard material collagen, the viability, intracellular lipids, and integrity of lipid vacuoles of ACs encapsulated in GG were evaluated (**Figure** **5**A). Live/dead staining of encapsulated ACs revealed evenly distributed viable ACs throughout the hydrogel for both matrix materials for 3 days (Figure 5C,D). In the cell cycle and cell turnover process, free lactate dehydrogenase (LDH) can be correlated with disrupted cell membrane integrity, implicating cell death. On day 1, after hydrogel formation, normalized LDH release from the ACs encapsulated in GG hydrogels (128.66% ± 26.20) and cultured in serum‐containing medium exhibited a significantly higher level than collagen (100% ± 17.27) (Figure 5C). In the defined medium, LDH levels of AC‐GG‐hydrogels were slightly elevated compared to collagen‐based hydrogels on day 1 (GG: 120.12% ± 26.85) (Figure 5D). LDH levels of ACs encapsulated in GG hydrogels (GG serum: 94.55% ± 16.26, GG defined: 103.98% ± 30) adapted to LDH levels of collagen‐encapsulated ACs (serum day 3: 100% ± 13.23, defined day 3: 100% ± 28.60) until day 3 in both media. This indicates a not significantly altered viability of ACs in GG and collagen hydrogels over the 3‐day analysis period and qualifies GG as a suitable material for AC encapsulation in this study. The metabolic activity measurements via WST assay revealed high variation for ACs in collagen‐based hydrogels in serum‐containing and defined medium on days 1 and 3. ACs encapsulated in GG‐hydrogels exhibited lower, but not significantly lower, metabolic activity than AC‐collagen gels (Figure 5C,D **metabolic activity)**.

Intracellular lipid staining revealed a uniform lipid accumulation across both types of hydrogels on days 1 and 3 of culture (Figure 5E,F). The BODIPY staining highlights a homogeneous distribution of intracellular lipid‐filled vacuoles across the GG hydrogel in both culture media for 3 days. Staining of the vacuole coating protein perilipin A confirmed the results of the lipid staining, with a continuous perilipin A coating surrounding the lipid vacuoles for all matrix materials and media conditions. ACs in collagen and GG hydrogels maintained their viability and lipid vacuoles for up to 7 days (Figure S4, Supporting Information). In addition to storing lipids, ACs can also metabolize and release them. The lipolytic activity was assessed by determining the degradation product glycerol in the cell culture supernatant. The released glycerol concentration was similar for the ACs in GG hydrogels on day 1 (59.55 ng mL^−1^ ± 12.64) compared to ACs encapsulated in collagen (65.55 ng mL^−1^ ± 8.11) in both media conditions (Figure 5E,F **glycerol release)**. On day 3, glycerol concentrations did not differ significantly for collagen‐ and GG‐based hydrogels in serum‐containing medium (col: 77.66 ng mL^−1^ ± 17.45, GG: 59.26 ng mL^−1^ ± 12.61) and in the defined medium (col: 55.23 ng mL^−1^ ± 26.32, GG: 44.91 ng mL^−1^ ± 10.22). Summarized, ACs encapsulated in 0.5% GG maintained functionality and exhibited similar viability and basal glycerol release to collagen for 3 days. ACs in GG hydrogels without monocytes were analyzed for cytokine release in serum‐containing and defined medium. Unstimulated and PMA, LPS, or PMA+LPS‐stimulated ACs in GG hydrogels released detectable amounts of IL‐6 and IL‐8, whereas IL‐1β and TNFα were below the detection limit (Figure S5, Supporting Information).

### 2.3 The 3D Adipocyte‐Macrophage Co‐Culture Exhibits High Viability and can be Induced into a Pro‐Inflammatory State

**Figure 6:**
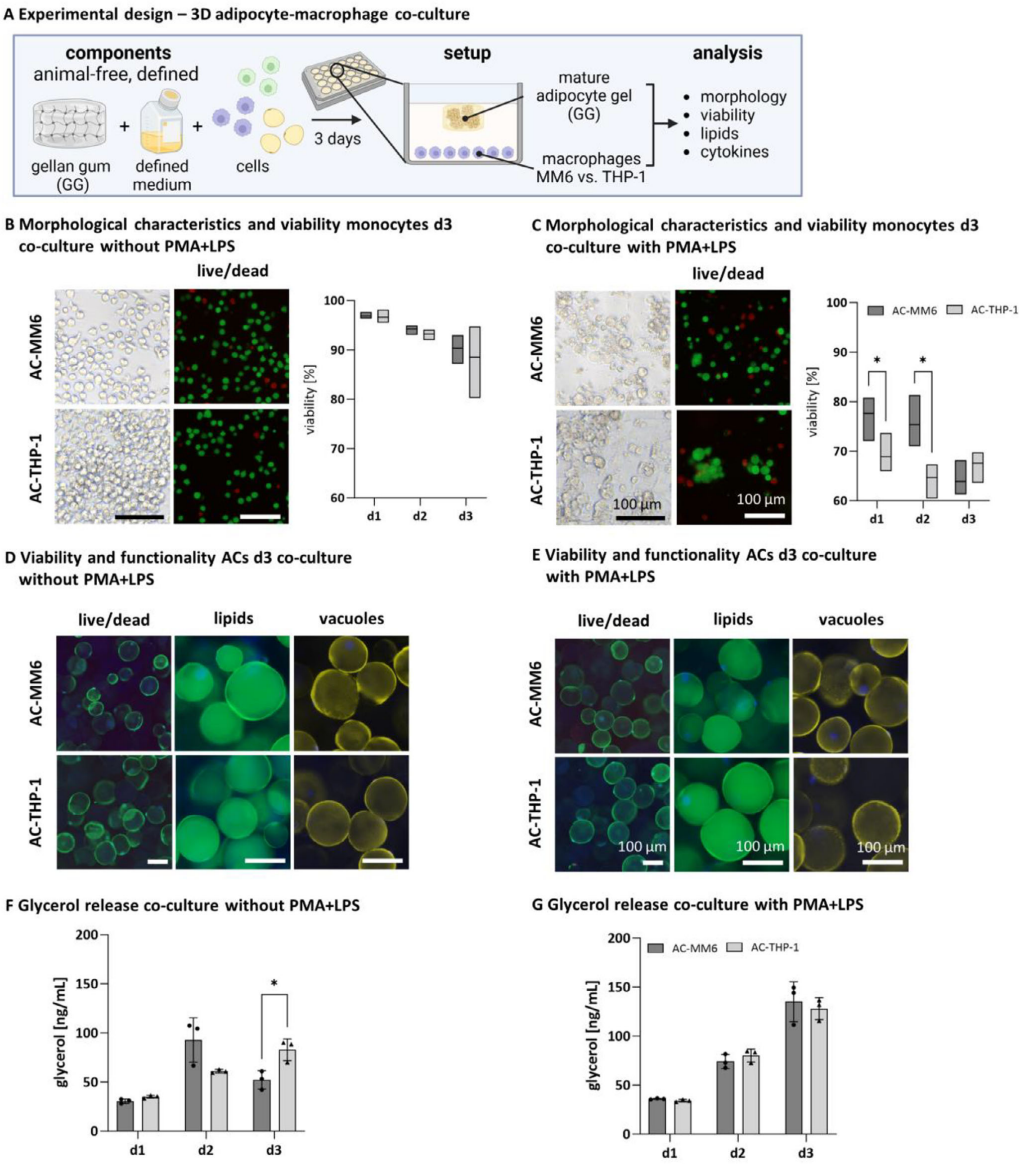
Viability, metabolic activity, and lipid content of the defined inflamed co‐culture after 3 days. A) Experimental design of the co‐culture setup with animal‐free matrix material in the defined medium. B) Phase contrast images and live/dead staining of unstimulated (without PMA+LPS) and C) stimulated (with PMA+LPS) monocytes in co‐culture on day 3, viable cells in green, dead cells in red. Scale bar 100 µm. Semi‐quantitative evaluation of the viability of MM6 and THP‐1 in co‐culture over 3 days. D) Live/dead and intracellular lipid (BODIPY) staining of unstimulated (without PMA+LPS) and E) PMA+LPS‐stimulated (with PMA+LPS) co‐cultured AC‐GG hydrogels, viable cells in green, dead cells in red, lipids in green, nuclei in blue. Scale bar 100 µm. F) Basal glycerol release of unstimulated and G) stimulated co‐culture in ng mL^−1^. Mean values represent the mean ± SD of three independent experiments with three different biological donors. ^*^ *p* ≤ 0.05.

The animal‐free and defined components (hydrogel material and culture medium) were combined into a co‐culture, using MM6/THP‐1 seeded in well plates and an AC‐GG hydrogel floating above for 72 h of culture (**Figure** **6**A). Both cell types, monocytes, and ACs, were evaluated for their viability in the co‐culture and were activated with PMA+LPS to induce an inflammatory state. An unstimulated co‐culture served as a negative control. Unstimulated and PMA+LPS‐activated monocytes in co‐culture were phenotypically evaluated by clumping assay. Phase contrast images of unstimulated MM6 and THP‐1 show roundish, non‐clumped cells and the activated cells (PMA+LPS) showed characteristic clumping and partial adherence on day 3 (Figure 6B,C). Overall, MM6 and THP‐1 viability was high in the unstimulated control, with few dead cells, visualized by live/dead staining. The semi‐quantitative evaluation revealed significantly lower viability (MM6: 63.86% ± 7.96, THP‐1: 67.59% ± 6.94) for PMA+LPS‐activated MM6 and THP‐1 compared to the unstimulated control (MM6: 90.35% ± 2.99, THP‐1: 88.54% ± 6.90) (Figure 6B,C). Encapsulated ACs in co‐culture were analyzed for their viability by live/dead staining and their functionality by visualizing intracellular lipids by BODIPY, lipid vacuole quality by perilipin A staining, and measurement of lipid turnover by glycerol release. ACs showed a uniform distribution of viable cells in the co‐culture, unaffected by stimulation and the type of monocytes co‐cultured with (Figure 6D,E **live/dead)**. Intracellular lipid staining revealed homogeneously distributed lipid‐filled cells for both co‐culture models with no visible alterations in lipid storage (Figure 6D,E **lipids**). Perilipin A integrity of the co‐culture was slightly impaired in the PMA+LPS‐stimulated condition, visible by a non‐uniform coating of the lipid vacuoles compared to the unstimulated control (Figure 6E **lipid vacuoles**). To determine the functionality of the ACs in the inflamed model, the basal glycerol release in the supernatant was measured over 72 h (Figure 6F,G). Basal glycerol measurements complemented the findings of the perilipin A staining for the AC‐MM6 co‐culture, as the lipolytic activity of the PMA+LPS‐stimulated group exceeded the unstimulated group on day 3 (unstimulated: MM6 day 3: 58.39 ng mL^−1^ ± 15.30, PMA+LPS: MM6 day 3: 134.27 ng mL^−1^ ± 20.60). For THP‐1, basal glycerol measurements confirmed the findings of the perilipin A staining with rising glycerol levels for PMA+LPS‐treated co‐culture over 3 days, indicating an elevated lipid breakdown. Glycerol values of stimulated AC‐THP‐1 co‐cultures exceeded the unstimulated control on day 3 of culture (unstimulated THP‐1 day 3: 81.75 ng mL^−1^ ± 9.94, PMA+LPS day 3: 125.90 ng mL^−1^ ± 13.74). Basal glycerol release did not significantly differ between AC‐MM6 and AC‐THP‐1 co‐cultures except for day 3 in the unstimulated control, with higher levels for THP‐1 co‐cultured models (unstimulated: MM6 day 3: 58.39 ng mL^−1^ ± 15.30, unstimulated THP‐1 day 3: 81.75 ng mL^−1^ ± 9.94). Additionally, morphological characteristics, viability, and basal glycerol release of co‐cultures stimulated with PMA or LPS only are shown in Figure S6 (Supporting Information).

**Figure 7:**
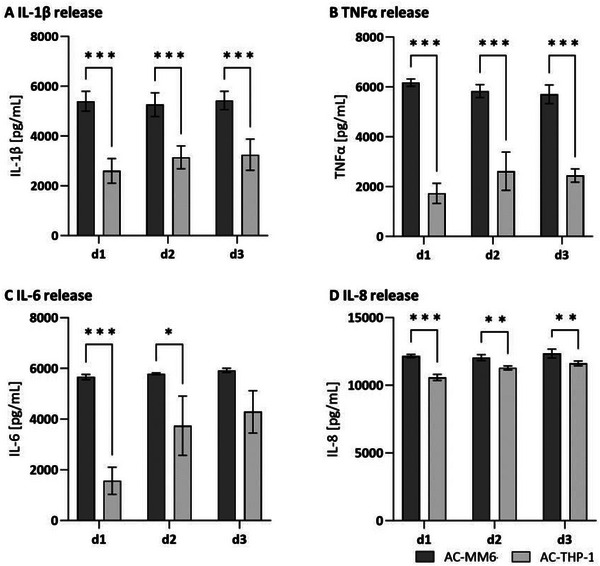
Cytokine release in co‐culture after 3 days. A) IL‐1β, B) TNFα, C) IL‐6, and D) IL‐8 in the supernatant of PMA+LPS‐activated co‐culture (AC‐MM6, AC‐THP‐1). Mean values represent the mean ± SD in pg mL^−1^ of three independent experiments with three different biological donors. ^*^ *p* ≤ 0.05, ^**^ *p* ≤ 0.01, ^***^ *p* ≤ 0.001.

The secretion of pro‐inflammatory cytokines of the defined co‐culture was quantified by ELISA over 3 days. After 72 h of activation, a significant cytokine response was detected compared to the unstimulated control for the co‐cultures activated with PMA+LPS throughout all conditions (**Figure** **7**). The PMA+LPS‐activated co‐culture with MM6 released nearly constant levels of the cytokines IL‐1β, TNFα, IL‐6, and IL‐8 (MM6 day 3: IL‐1β 5429.21 pg mL^−1^ ± 387.90, TNFα 5704.25 pg mL^−1^ ± 389.00, IL‐6 5923.13 pg mL^−1^ ± 197.27, and IL‐8 12340.99 pg mL^−1^ ± 309.27) of the inflammatory panel over 3 days. AC‐THP‐1 co‐cultures showed varying cytokine profiles with increasing values from day 1 to day 3 for IL‐1β, IL‐6, and IL‐8 (THP‐1 day 3: (THP‐1 day 3: IL‐1β 3248.60 pg mL^−1^ ± 520.64, IL‐6 4287.23 pg mL^−1^ ± 807.42, and IL‐8 11620.23 pg mL^−1^ ± 191.76) and for TNFα from day 1 to day 2 (THP‐1 day 2: 2614.94 pg mL^−1^ ± 714.29). Co‐cultures with MM6 exhibited higher and more constant cytokine levels for IL‐1β, IL‐6, and TNFα and similar levels for IL‐8 over 3 days compared to those with THP‐1. Cytokine release for co‐cultures stimulated with PMA or LPS is shown in Figure S7 (Supporting Information).

## 3 Discussion

In the present study, we established an animal‐free adipocyte‐macrophage co‐culture to model inflamed adipose tissue in vitro, yielding rapid results within 72 h. This approach facilitates the development of a more reliable in vitro test system for quick results that avoids undefined composition and batch‐to‐batch variations, as serum components and animal‐derived matrix materials can distort results.^[^ ^31^, ^40^ ^]^ PMA+LPS stimulation successfully activated the co‐culture to an inflammatory state with distinct cytokine profiles for MM6‐ and THP‐1‐containing co‐cultures.

### 3.1 Evaluation of the Components for the Inflamed 3D Adipocyte‐Macrophage Co‐Culture

When designing in vitro models of inflamed adipose tissue, it is crucial to consider the cell source (primary or cell line) and type (human or other species). ASCs are commonly used for adipose tissue engineering; however, they must be differentiated before applying the in vitro model.^[^ ^13^ ^]^

In this study, we used primary human ACs to construct a simplified 3D co‐culture with an immune component in vitro. ACs offer the advantage of being already terminally differentiated and resembling the physiological state of functional ACs, ready to be used in the co‐culture without previous differentiation.^[^ ^11^ ^]^ Using ACs in in vitro models also offers the potential for incorporating patient‐derived cell material for personalized approaches. A study by Keuper introduced a system of a human monocytic cell line and a preadipocyte cell line, either indirectly or directly co‐cultured, and demonstrated expected characteristics regarding insulin sensitivity in an inflammatory environment.^[^ ^41^ ^]^ Others have used an adipocyte (3T3‐L1) and a macrophage cell line (C2D) of murine origin to study the crosstalk of adipocytes and macrophages.^[^ ^5^ ^]^ Differences between human and murine adipocytes and monocytes result in limited transferability of murine to human studies and need verification for the human system.^[^ ^42^ ^]^ Thus, the primary human ACs and monocytic cell lines MM6 and THP‐1 used in this study apply to a more human‐like inflamed adipocyte‐macrophage co‐culture.

In adipose tissue dysfunction, the inflammatory reaction involves various immune cell types, with macrophages being the most abundant immune component that elicits pro‐inflammatory cytokines.^[^ ^43^ ^]^ The absence of other immune cells, such as T‐cells, dendritic cells, or crosstalk partners like fibroblasts, in this co‐culture was intended to maintain focus on the macrophage‐adipocyte interaction and to keep the complexity at a moderate level. The human monocytic cell lines were chosen for their availability and compared as components of the co‐culture due to their different origins and reactions to stimulation reported in the literature.^[^ ^44^ ^]^

The monocytic cell lines MM6 and THP‐1 were tested for their activation potential by stimulation with PMA+LPS in serum‐containing and our in‐house developed defined medium. LPS is a component of gram‐negative bacteria and a naturally occurring immune‐activating agent.^[^ ^45^ ^]^ PMA is a defined, synthetic factor already used to differentiate monocytes in vitro. THP‐1 monocytes partially adhere and aggregate upon stimulation with PMA.^[^ ^15^, ^46^, ^47^ ^]^ This behavior was also observed for the MM6 in this study.

Besides an artificial induction by bacterial components or synthetic stimulants, obesity‐related inflammation could be triggered by free fatty acids.^[^ ^16^ ^]^ For a more physiological setup, multifactorial triggers such as free fatty acids could be added to consider their overload during obesity (lipotoxicity) to effectively mimic the complex condition of adipose tissue dysfunction in vitro. The group of Pieters treated macrophages with fatty acid‐stimulated adipocyte‐conditioned media and found some influence of palmitic acid on IL‐6 and TNFα expression levels, although the cytokine expression levels of treated adipocytes were not significantly altered in this setup.^[^ ^48^ ^]^ Others have found that free fatty acids stimulate an inflammatory expression profile in macrophages.^[^ ^49^ ^]^ The presented study focused on an animal‐free setup and eliciting a strong inflammatory reaction in the co‐culture to challenge the cells for measurable secretory responses. To further investigate the inflammatory response of the MM6 and THP‐1 to the exogenous stimuli (LPS+PMA), the pro‐inflammatory cytokines IL‐1β, IL‐6, IL‐8, and TNFα were measured. The cytokine levels in the serum‐containing and defined media were comparable per cell line but differed between them. We observed varying results for LPS and PMA stimulation only, indicating that the activation capacity differs between stimulants, and not every activation pathway is triggered with one stimulant alone.

In our study, we used constant concentrations of PMA and LPS for the stimulation of the co‐culture, which was based on values from a literature review.^[^ ^35^, ^50^, ^51^ ^]^ The treatment protocol could be further optimized by titration of each stimulant, as, amongst other factors, other groups have shown that the concentration of PMA influences the differentiation of THP‐1.^[^ ^52^ ^]^ The combined stimulation with PMA+LPS led to the highest cytokine concentrations across all measured cytokines in both cell lines compared to PMA or LPS stimulation alone. It was also observed by the group of Liu that PMA‐treated THP‐1 showed an increased sensitivity to LPS, eliciting strong reactions.^[^ ^53^ ^]^

This study highlights the influences of the in vitro model setup and the use of the activation stimuli PMA and LPS, or their combination, on inducing the secretion of pro‐inflammatory cytokines in the defined adipocyte‐macrophage co‐culture. The characterization of inflammation focuses on the secretory reaction of the co‐culture, and incorporating molecular analyses could provide deeper insights into the impact of the involved signaling pathways.

The group of Kim studied the activation of signaling pathways of PMA‐differentiated THP‐1 and non‐differentiated THP‐1 cells in response to LPS treatment. They found more LPS‐related receptor proteins (toll‐like receptor 4) and higher phosphorylation levels for mitogen‐activated protein kinase (MAPK) and nuclear factor kappa‐light‐chain‐enhancer of activated B‐cells (NF‐κB) pathway‐related molecules, demonstrating a higher susceptibility of PMA‐differentiated THP‐1 cells to LPS.^[^ ^50^ ^]^ In another study, the group of Liu proved activation of downstream signals of the NF‐κB and signal transducer and activator of transcription 3 (STAT3) pathways in LPS‐treated THP‐1.^[^ ^53^ ^]^ Given the multifactorial triggers for obesity‐related inflammation, various signaling pathways such as NF‐κB, MAPK, and STAT are involved in metabolic stress and immune activation.^[^ ^54^ ^]^

In summary, it was possible to activate MM6 and THP‐1 by PMA+LPS stimulation in the defined medium, as measured by the secretome. Both monocytic cell lines were used and compared to establish the inflamed 3D adipocyte‐macrophage co‐culture.

In addition to the cell source, selecting a suitable animal‐free matrix material is essential.

Currently, several animal‐free matrix materials exist for constructing in vitro adipose tissue models, such as GG, alginate, hyaluronan, or polyethylene glycol.^[^ ^23^, ^24^, ^25^, ^55^ ^]^ The choice of the ideal animal‐free matrix material depends on the required properties for specific research applications and practical considerations, such as cell compatibility, mechanical stability, or availability. GG is particularly suited for defined in vitro adipose inflammation models due to its mild gelation conditions and tunable stiffness to mimic soft tissue. Unlike PEG, alginate, or hyaluronan, it provides structural stability without chemical modification and minimizes immune interference.

As previously demonstrated by our group, GG was selected as a tunable and biocompatible material for adipose tissue culture.^[^ ^27^ ^]^ There was also no activation through the matrix material, as we have already tested GG to ensure it does not influence monocyte activation.^[^ ^27^ ^]^ ACs were mixed into GG solutions with varying concentrations (0.5–1.0%) to evaluate GG for the AC hydrogel setup in co‐culture. As ACs are very sensitive to mechanical stress, the mixing behavior of the cell suspension into the GG solution was evaluated.^[^ ^56^ ^]^ At higher GG concentrations (above 0.7%), areas of increased material without the yellowish cell suspension are visible (Figure S3, Supporting Information). This can be explained by the higher polysaccharide concentration, which is associated with this; the viscosity increases, the gelling temperature decreases, and the pore size of the network becomes smaller.^[^ ^57^ ^]^ The faster the GG solution gets solid and the smaller the pores are, the more difficult it is to mix in ACs.

It is worth mentioning that the two materials have different sol–gel transition points, as collagen gels with increasing temperature and GG with decreasing temperature. The gelation in collagen is based on the temperature increase and a pH shift.^[^ ^58^ ^]^Within a polysaccharide, such as GG, gelation takes place due to the enhanced interactions of the carbohydrate molecules with each other.^[^ ^29^ ^]^ The fact that the GG solution is not fully crosslinked while mixing with ACs helps reduce the mechanical force during the encapsulation. The AC‐GG hydrogels are stable, despite a culture temperature above the sol–gel transition, due to the crosslinking with divalent ions present in the cell culture medium. As 0.5% GG concentration shows the best processing and the softest matrix, it was further used in the co‐culture.

In adipose tissue engineering, collagen is widely used as a matrix material.^[^ ^13^, ^59^ ^]^ Therefore, we tested if the selected 0.5% GG matrix is comparable to collagen in terms of the presence of intracellular lipids, as well as viability via LDH release and lipolytic rate via glycerol release. Lipid‐filled cells were visible in both matrix materials until day 7 of culture (Figure S4, Supporting Information). The ACs were more densely packed in the GG hydrogels, comparable to the native lobule structure of adipose tissue, and not as dispersed as in the collagen hydrogels. For the LDH release for GG hydrogels, the highest values were detected on day 1 compared to collagen. This could be caused by the higher viscosity of GG during processing, leading to a higher resistance while mixing and potentially impairing the cells. Nevertheless, LDH release normalizes until day 3 of the culture of ACs encapsulated in GG, and glycerol concentrations were not significantly altered compared to collagen‐encapsulated ACs. This demonstrates that GG is a suitable material for the engineering of the 3D co‐culture in this study, which aligns with the findings of other groups.^[^ ^21^, ^60^ ^]^

A defined medium we developed in‐house was tested for AC culture as a later option for co‐culture. The GG‐encapsulated ACs remained viable and exhibited a high content of lipid‐filled vacuoles over 3 days in the defined medium. The slightly increased glycerol release could be due to the GG hydrogel processing and general lipid turnover.^[^ ^61^ ^]^ Others have shown that differentiation and maintenance of ASCs in combination with endothelial cells is possible in a completely defined in vitro environment.^[^ ^62^ ^]^ Thus, we tested our defined medium and demonstrated that it is suitable for AC culture and the following co‐culture experiments, offering a more relevant in vitro system.

### 3.2 Setup of the Defined Inflamed Adipose Tissue‐Like Test System

After proving the activation potential of the MM6 and THP‐1 in the defined medium and selecting GG as an animal‐free matrix material for AC culture, the animal‐free and defined co‐culture was set up. Both co‐cultures were successfully activated by PMA+LPS stimulation, determined by the release of inflammatory cytokines IL‐1β, IL‐6, IL‐8, and TNFα. The group of Lund observed an influence on cytokine release by varying PMA withdrawal times and found decreasing TNFα levels with increasing resting times after PMA stimulation.^[^ ^46^ ^]^We treated the co‐culture with PMA+LPS simultaneously for 72 h and detected the highest cytokine levels compared to PMA or LPS stimulation alone. Controversially, cytokine response for IL‐1β and TNFα was barely detectable after stimulation with LPS only (Figure S7, Supporting Information). This is contrasted by the findings of others, which have demonstrated that LPS potently stimulates monocytes to release inflammatory cytokines in vitro.^[^ ^63^ ^]^ Our findings suggest a varying stimulation capacity of LPS, possibly due to batch‐to‐batch variations.^[^ ^64^ ^]^ Environmental factors and the stimuli added strongly influence the characteristics of macrophages, as they are a dynamic cell population.^[^ ^65^ ^]^ This is also true for MM6 and THP‐1, as the nature and different concentrations of activating stimuli can alter the cytokine release of these cells.^[^ ^66^ ^]^ The variances found in LPS activation highlight the importance of defined activation conditions and consistent protocols to increase the comparability of results, which was aimed at in this study.

Another group also investigated the effect of different PMA stimulation protocols on the differentiation of the monocytic cell line THP‐1.^[^ ^47^ ^]^ Moreover, the group of Traore demonstrated that PMA treatment induced growth inhibition in the monocytic cell line THP‐1.^[^ ^67^ ^]^ MM6 also showed growth inhibition upon PMA+LPS activation in our study.

Basal glycerol release of PMA+LPS‐treated models increased significantly on day 3 compared to unstimulated models, indicating an impact of the activation factors on lipid metabolism. There are similarities with findings from the group of Grisouard, as they prove a correlation between LPS stimulation and the induction of lipolysis in differentiated human ASCs.^[^ ^68^ ^]^ However, in our model, LPS stimulation alone did not significantly alter the basal glycerol release compared to the unstimulated control. For PMA, the group of Fricke investigated the influence of PMA stimulation on glycerol release of differentiated human ASCs. They found a dose dependence of glycerol levels with increasing PMA concentration.^[^ ^69^ ^]^ This aligns with our findings for our adipose tissue‐like model on day 3, where PMA and PMA+LPS triggered a higher glycerol release than unstimulated and LPS‐treated models. Basal glycerol levels were measured in this study to point out the direct effect of the stimulants on glycerol release for basic characterization. Glycerol release stimulated by isoproterenol, a β‐adrenergic agonist, could be used to further investigate the influence of inflammation on lipid metabolism and complement results as a functional assay.^[^ ^70^ ^]^

Inflammatory processes can induce morphological changes in the affected cells.^[^ ^7^, ^39^ ^]^ Therefore, the inflamed adipose tissue‐like model was further characterized by investigating the influence of activation factors on perilipin A expression of ACs by fluorescence staining. Perilipin A is a coating protein found exclusively on the surface of lipid storage droplets in adipocytes and modulates lipid storage and mobilization.^[^ ^71^ ^]^ In this study, partially inconsistent perilipin A covering of the lipid vacuole of ACs treated with PMA+LPS was observed. This aligns with other studies demonstrating that perilipin A integrity is influenced by activation factors like PMA, stimulating signaling pathways that result in the phosphorylation of perilipins, leading to their degradation and lipid release.^[^ ^69^, ^72^ ^]^ Additionally, obesity‐related inflammatory mediators like TNFα have been shown to reduce perilipin expression in ACs.^[^ ^73^ ^]^

The inflamed 3D adipocyte‐macrophage co‐culture is an easy‐to‐assemble and animal‐free co‐culture for studying early inflammatory processes. Using mature ACs and the human monocytic cell lines MM6 or THP‐1 enables a setup in 3 days for modeling adipose‐related inflammatory processes in vitro. Cell‐line‐based models can mirror the characteristics of ATMs by adopting inflammatory phenotypes, as the group of Bhatia demonstrated with a THP‐1 and 3T3‐L1 co‐culture.^[^ ^36^ ^]^

To study adipose tissue in health and disease, on‐chip models and perfusion systems have been developed.^[^ ^38^, ^74^ ^]^ Most studies use ASCs or differentiated mesenchymal stem cells, and only a few use mature ACs due to their buoyancy and fragility in culture.^[^ ^37^, ^75^ ^]^The model from Rogal incorporated mature ACs, including other adipose tissue‐associated cells like endothelial cells and PBMCs, into a chip device with media perfusion. They analyzed the adipose tissue model in the chip over 13 days, demonstrating its responsiveness to inflammatory stimuli and almost fully resembling in vivo tissue composition.^[^ ^37^ ^]^ We successfully established the use of mature ACs encapsulated in GG hydrogels in our model, as they offer the advantage of being ready to use after isolation without time‐consuming differentiation and determining differentiation status. Moreover, our model was cultured in a defined environment, eliminating serum‐related influences and animal‐derived matrix materials and increasing the relevance of the in vitro test system. Considering the extended culture periods of other groups for adipose tissue models, one limitation of the presented co‐culture model may be the restricted culture period of 3 days due to the proliferation of MM6 and THP‐1.^[^ ^76^ ^]^ To investigate the long‐term effects of inflammation in order to mimic more chronic conditions and increase the physiological relevance of the model, a prolonged culture time should be achieved by further optimizing the co‐culture, for example, by integrating primary monocytes into the hydrogel. Nevertheless, up to 72 h of culture enables the investigation of acute inflammatory responses and direct effects of inflammation on the adipose cell model. The 3D adipocyte‐macrophage co‐culture developed in our study works in a completely animal‐free and defined environment. It can be activated by PMA+LPS to a pro‐inflammatory state with robust cytokine secretion within 72h, simulating short‐term adipose tissue inflammation.

## 4 Conclusion

We developed a fully defined and easy‐to‐assemble 3D adipocyte‐macrophage co‐culture to study inflammation in vitro. Utilizing mature human adipocytes enables a ready‐to‐use setup while combining AC‐laden GG hydrogels and monocytes in a defined medium promotes cell viability, lipid retention, and pro‐inflammatory cytokine secretion. This model provides a controlled and reproducible platform for investigating the inflammatory secretome within 72 h and offers potential for preclinical drug testing. In the future, it could be adapted for patient‐specific disease models, facilitating personalized approaches to studying metabolic inflammation and assessing targeted therapies.

## 5 Experimental Section

### Monocyte Culture

The human acute monocytic leukemia cell lines Mono‐Mac‐6 (MM6, DSMZ Leibnitz Institute ACC 124) and THP‐1 (DSMZ Leibnitz Institute ACC 16) were cultured in serum‐containing medium RPMI‐1640 (Biowest, Nuaillé, France) [10% FCS (PAN Biotech, Aidenbach, Germany), 1% oxaloacetate, pyruvate, insulin (OPI) supplement (Sigma–Aldrich, Steinheim, Germany), 1% MEM‐non‐essential amino acid solution (NEAA, PAN Biotech), 1% penicillin‐streptomycin (P/S, PAN Biotech) for MM6, or only 10% FCS and 1% P/S for THP‐1] or a newly developed defined medium (PELOBiotech, Planegg, Germany) in 24‐well plate format (Greiner Bio‐One, Frickenhausen, Germany) at a cell density of 0.5 × 10^6^ cells in 1.2 mL at 37 °C, 5% CO_2_ for up to 7 days.

The defined medium was developed in cooperation with PELOBiotech. It consists of a basal medium (endothelial cell basal medium Cat.No. PB‐BH‐100‐9896) with glutamine, human serum albumin, and P/S + a defined supplement mix [recombinant human growth factors and hormones, ascorbic acid‐2‐phosphate, biotin, pantothenate, all PELOBiotech]. All components of the defined medium are available at PELOBiotech.

### Isolation and Culture of Mature Adipocytes

Human subcutaneous white adipose tissue biopsies were provided by Dr. Ziegler and the team (Robert Bosch Krankenhaus, Stuttgart, Germany). All research was carried out following the Declaration of Helsinki on human medical research. Patients gave written consent after being given information about using their samples. This is under the permission of the Landesärztekammer Baden‐Württemberg (F‐2012‐078; for normal skin from elective surgeries). ACs were isolated according to a previously described protocol.^[^ ^77^ ^]^ Briefly, fat lobules were dissected from the adipose tissue sample, cut into small pieces with tissue scissors, and digested with collagenase I NB4 (Nordmark Pharma, Uetersen, Germany) solution [1% bovine serum albumin (BSA, fraction V, Serva Electrophoresis, Heidelberg, Germany), 100 Units collagenase in Dulbecco's modified Eagle medium (DMEM, PAN Biotech)] for 1.5 h at 37 °C with light agitation. The digested tissue was sieved, and several washing steps with DMEM without phenol red followed. The freshly isolated ACs were allowed to settle for 1 h in DMEM F12 with 10% FCS and 1% P/S to inactivate collagenase residues, washed with PBS+ (Capricorn Scientific, Ebsdorfergrund, Germany), and immediately used for further experiments. Demographics for all tissue donors can be found in Table S1 (Supporting Information).

### Encapsulation of Adipocytes in Collagen and Gellan Gum

Freshly isolated ACs were encapsulated into collagen type I hydrogels (10 mg mL^−1^, rat tail, Corning, New York, USA) or gellan gum (0.5–1.0% w/v, Gelzan^TM^ CM, Sigma–Aldrich, Steinheim, Germany). For collagen‐based hydrogel formation, the AC suspension and collagen were mixed with a gel neutralization solution [10X DMEM/Ham's F12 (Biochrom, Cambridge, UK) and 50 mм NaOH in demineralized water (1:1) with 0.2 м NaHCO_3_ and 0.225 м HEPES (Serva Electrophoresis)] in a ratio of 4:4:1. One gel was composed of 60 µL collagen, 60 µL AC suspension and 15 µL gel neutralization solution. For one gel, 135 µL was pipetted into a plastic mold (8 mm diameter) and allowed to gel for 20 min at 37 °C. After incubation, the hydrogels were carefully detached from the molds with sterile forceps, and a serum‐containing maintenance medium (AM‐1, Amsbio, Abingdon, UK) or the newly developed defined medium (PELOBiotech) was added.

For GG‐based hydrogels, GG was dissolved in double‐distilled water to 0.5–1.0% w/v by boiling in a microwave until the solution was clear. The GG solution was tempered to 37 °C and was mixed with the AC solution. One gel was composed of 75 µL of the respective GG concentration and 60 µL of the AC suspension. 135 µL of the mixture per gel was pipetted into plastic molds, and PBS containing divalent ions was pipetted beneath and on the gels. After 10 min at 37 °C, the molds were removed carefully with sterile forceps. The adipocyte gels were cultured in 1.2 mL serum‐containing maintenance medium or defined medium in 24‐well plates for up to 7 days at 37 °C, 5% CO_2_.

### Rheological Analysis

A rotary oscillating rheometer (Kinexus, Malvern Panalytical, Worcestershire, UK) with a 1° cone‐plate and 60 mm diameter geometry was used to measure the temperature‐dependent sol–gel transition of the non‐crosslinked collagen and gellan gum mixtures. It was measured by oscillation at a constant frequency of 0.1 Hz and a shear stress of 0.1 Pa with a sample interval of 2 s. Measurements were performed at a temperature range from 50–10 °C (for GG) and 10–50 °C (for collagen), each with a ramp rate of 2.5 °C min^−1^.

### Co‐Culture of Adipocytes and Macrophages

The co‐culture was set up by adding AC‐GG hydrogels (60 µL AC suspension per gel) to 0.5 × 10^6^ monocytes in 1.2 mL defined medium in a 24‐well plate. AC hydrogels float above the seeded monocytes. Activation supplements were added to the respective medium, and treated cells were incubated for 72 h at 37 °C, 5% CO_2_. Untreated cells served as the negative control. The activation factors PMA (PELOBiotech) and LPS (*Escherichia coli* O55:B5, Sigma–Aldrich) were used at concentrations of 12.3 ng mL^−1^ and 1 µg mL^−1^, respectively. Supernatants for further cytokine analysis were taken after 24, 48, and 72 h of culture.

### Live/Dead Staining

For live/dead staining of ACs in gels and monocytes, the staining solution was prepared according to the manufacturer's instructions (LIVE/DEAD^TM^ Viability/Cytotoxicity Kit, Invitrogen, Thermo Fisher Scientific, Waltham, USA): 1 mL PBS+ was supplemented with 0.5 µL calcein, 2 µL ethidium homodimer, and 1 µL Hoechst 33342 and incubated for 1 h at room temperature (RT) in the dark. Microscopic analysis was done with an Axio Observer microscope and an Axiocam 305 color camera using ZENblue software (Carl Zeiss, Oberkochen, Germany). For image quantification, five representative images per donor and condition were counted using ImageJ to determine the number of viable and dead cells of MM6 and THP‐1 in the co‐culture semi‐quantitatively. The percentage of living monocytes was calculated from the live/dead cells ratio.

### Fluorescence Staining

Intracellular lipids were stained with BODIPY 493/503 dye (Cayman Chemicals, Ann Arbor, USA). Tissue samples were fixed with 4% paraformaldehyde (Roti®Histofix, Carl Roth, Karlsruhe, Germany) for 3.5 h and washed with PBS+. Fixed gels were incubated with staining solution [1 mL PBS+ supplemented with 1 µL BODIPY (1 µg mL^−1^) and 1 µL Hoechst (1 µg mL^−1^)] for 1 h at RT in the dark. Following two washing steps with PBS+ for 15 min.

For perilipin A staining, fixed GG‐AC gels were permeabilized for 30 min at RT in a permeabilization solution [0.1% Triton‐X (Sigma–Aldrich) in PBS+]. Permeabilized gels were blocked with blocking solution [3% BSA, 0.1% Triton‐X in PBS+] for 30 min and washed three times for 10 min with washing buffer [0.1% Tween in PBS+]. The primary antibody, rabbit anti‐human perilipin A (Sigma–Aldrich, P1998), was diluted 1:500 in blocking solution and incubated overnight at 4 °C. After three washing steps, the secondary antibody, goat anti‐rabbit IgG‐Alexa488 (Abcam, Cambridge, UK, ab60314), was diluted 1:500 in blocking solution and incubated for 30 min at RT in the dark. Gels were washed and counterstained with 4’, 6‐diamidino‐2‐phenylindole (DAPI, 1µg mL^−1^, Serva Electrophoresis) for nuclei staining. After three washing steps, the gels were stored in PBS+ at 4 °C until microscopic examination.

### Metabolic Activity (WST)

The metabolic activity of the AC gels was assessed by a WST assay. Cells were incubated with 1 mg mL^−1^ WST‐1 solution (TaKaRa Bio Europe, Saint‐Germain‐en‐Laye, France) in phenol red‐free DMEM medium for 3 h at 37 °C, 5% CO_2_. WST‐1 reduction was quantified by measuring the absorption at 450 and 620 nm (reference wavelength) using a microplate reader (SpectraMax i3, Molecular Devices, San Jose, USA).

### Lactate Dehydrogenase Assay

The release of LDH in the cell culture supernatant was determined by an LDH assay kit (Roche, Basel, Switzerland). Cell culture supernatant and the LDH reagent (1:50 catalyst: dye, 25 µL catalyst + 1.125 mL dye) were mixed 1:1 (50 µL + 50 µL per well) in a 96‐well plate. The plate was incubated for 30 min at RT in the dark and measured at 480 nm with a wavelength correction set at 680 nm with a SpectraMax i3 (Molecular Devices).

### Glycerol Assay

The glycerol release was quantified by an assay kit (Randox Laboratories, Crumlin, Ireland) according to the manufacturer's instructions. Cell culture supernatant was diluted in the provided assay buffer (1:1.57, 35 µL sample + 20 µL buffer), and a standard series in buffer (0–100 ng mL^−1^, 55 µL per well) was prepared in a 96‐well plate. 100 µL per well of the glycerol reagent was added to the diluted samples and incubated for 10 min at RT in the dark. Samples were measured in duplicates in a 96‐well plate at 520 nm with a SpectraMax i3 (Molecular Devices).

### Quantification of Extracellular Cytokine Release

To investigate the activation of mono‐ and co‐cultures, the cytokines IL‐1β, IL‐6, IL‐8, and TNFα were quantified by sandwich enzyme‐linked immunosorbent assay (ELISA, PeproTech, Thermo Fisher Scientific). ELISAs were carried out according to the manufacturer's instructions. Samples were diluted with diluent [0.05% Tween‐20, 0.1% BSA in PBS‐] (**Table** **1**). For substrate reaction, 100 µL of 2,2'‐azino‐bis(3‐ethylbenzothiazoline‐6‐sulfonic acid) (ABTS, Sigma–Aldrich) was added and incubated for 10–30 min. The absorption was measured at 10‐min intervals at 450 nm with a wavelength correction of 620 nm with a SpectraMax i3 (Molecular Devices).

**Table 1:** Dilution factors for ELISA samples.

Cytokine	Sample	Dilution factor
IL‐1β	Unstimulated, PMA, LPS, PMA+LPS	Undiluted
TNFα	Unstimulated, PMA, LPS PMA+LPS	Undiluted 1:2
IL‐6	Unstimulated, PMA, LPS PMA+LPS	Undiluted 1:2
IL‐8	Unstimulated, PMA, LPS PMA+LPS	1:4 1:10

### Statistics

Data were obtained from three independent experiments involving three distinct cell batches for the monocytic cell lines and three different biological donors for the mature ACs. All values are shown as mean ± standard deviation. Data were analyzed for significance by two‐way analysis of variance (ANOVA) followed by a posthoc test (Bonferroni). Statistics were conducted with GraphPad Prism 10.1.2. Results were stated as statistically significant when ^*^ *p* ≤ 0.05, ^**^ *p* ≤ 0.01, or ^***^ *p* ≤ 0.001.

## Conflict of Interest

The authors declare no conflict of interest.

## Author Contributions

S.No., S.N., F.B.A., and P.J.K. contributed to the conceptualization of the study. S.No. developed the methodology, performed the validation, conducted the investigation, carried out the formal analysis (along with F.B.A.), prepared the original draft, and created the visualizations. P.J.K. provided the resources, supervised the project (along with S.N.), administered the project, and acquired the funding. All authors contributed to the review and editing of the manuscript. All authors read and approved the final version of the manuscript.

## Supporting information



Supporting Information

## Data Availability

The data that support the findings of this study are available from the corresponding author upon reasonable request.
