# Supplementary material for: Animal‐Free Setup of a 3D Mature Adipocyte‐Macrophage Co‐Culture to Induce Inflammation In Vitro
Source: Adv Healthc Mater. 2025 Jun 20;14(22):2500779. doi: 10.1002/adhm.202500779 (PMC12391635; doi:10.1002/adhm.202500779)
Supplement: Supplementary file 1 — Supporting Information [file ADHM-14-0-s001.docx]

Supporting Information

**Animal-Free Setup of a Three-Dimensional Mature Adipocyte-Macrophage Co-Culture to Induce Inflammation *in vitro***

Sophia Nowakowski, Svenja Nellinger, Franziska Brigitte Albrecht, and Petra Juliane Kluger*


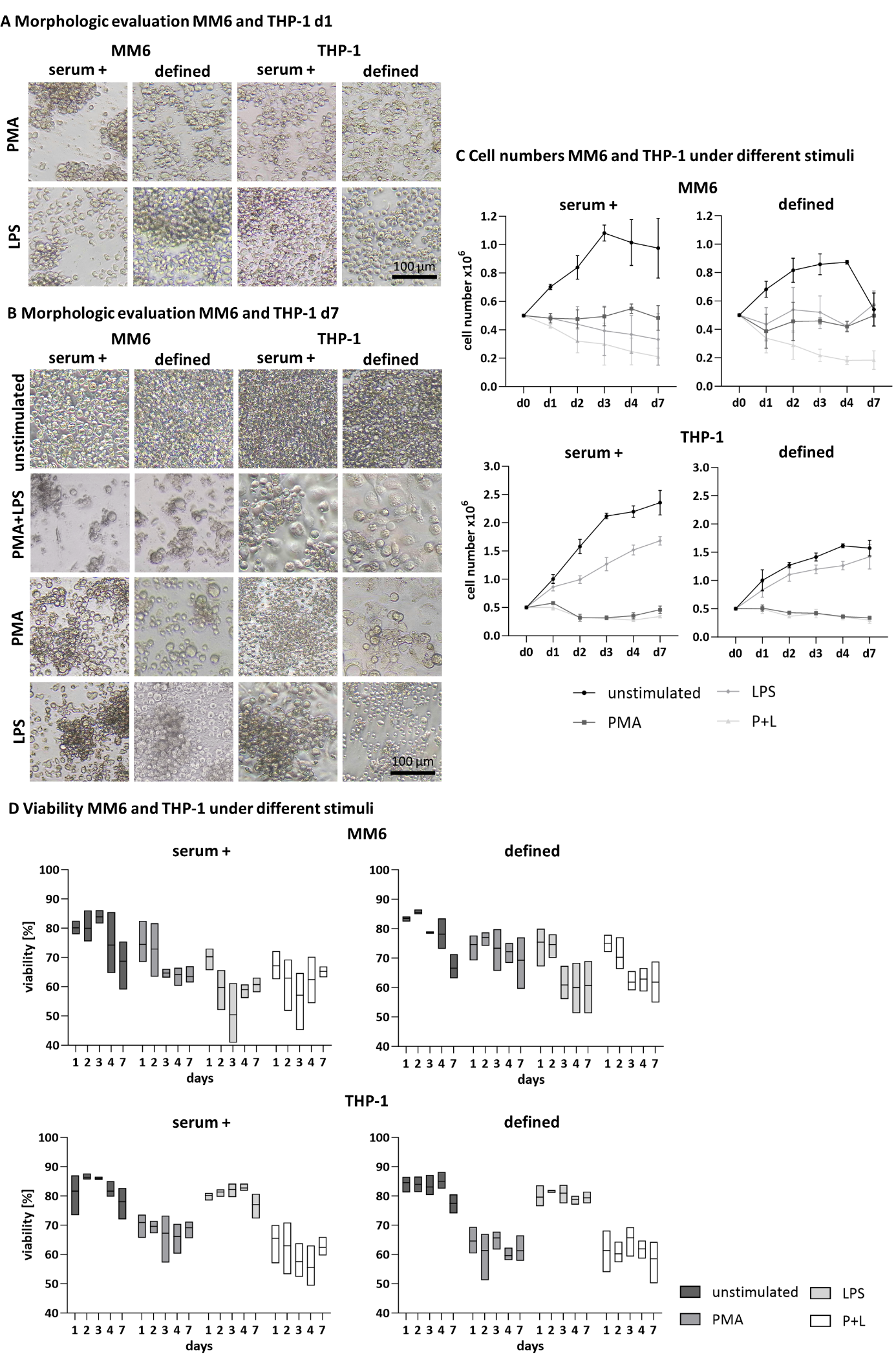


**Figure S1.** Monocyte (MM6 and THP-1) activation in serum-containing and defined medium in monoculture over 7 days. A) Phase contrast images of MM6 and THP-1 activated by PMA or LPS in the serum-containing medium (serum +) and the defined medium (defined) on day 1, and B) phase contrast images of unstimulated MM6 and THP-1 or activated by PMA, LPS, or PMA+LPS on day 7. Scale bar 100 µm. C) Cell numbers of MM6 and THP-1 over 7 days in the respective medium. Cell numbers are displayed as x10^6^ cells. D) Viability [%] of unstimulated and stimulated MM6 or THP-1 over 7 days in the respective medium. Mean values represent the mean ± SD of three independent experiments with distinct cell batches.


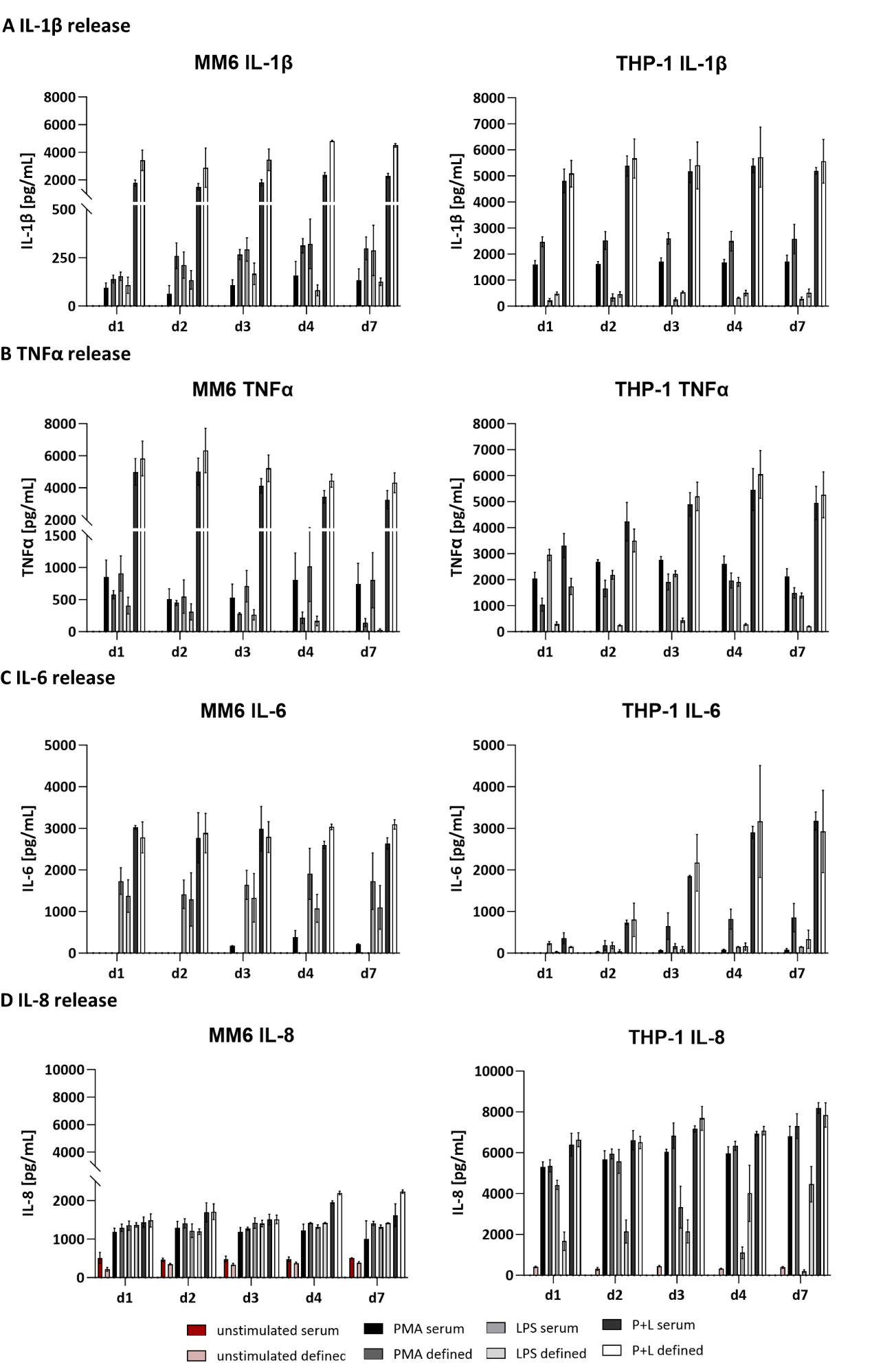


**Figure S2.** Release of cytokines A) IL-1β, B) TNFα, C) IL-6, and D) IL-8 of unstimulated and PMA, LPS or PMA+LPS-activated MM6 and THP-1 in serum-containing and defined medium over 7 days. Mean values represent the mean ± SD in pg mL^-1^ of three independent experiments with distinct cell batches.


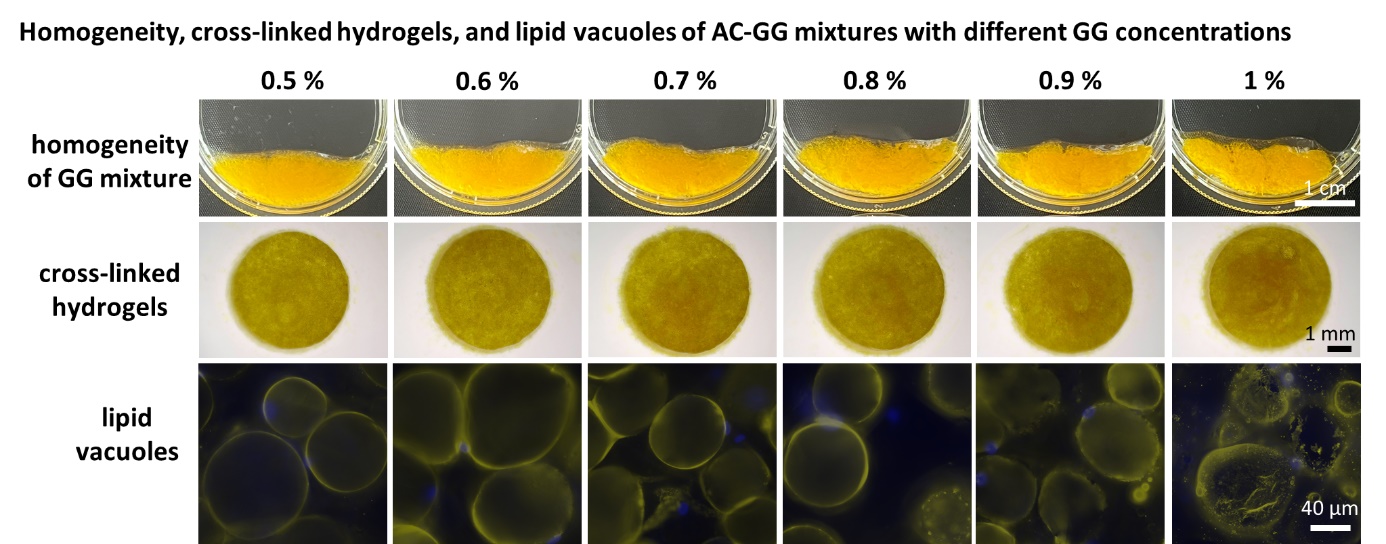


**Figure S3.** Homogeneity of AC-GG mixtures, cross-linked hydrogels, and lipid vacuoles of AC-GG hydrogels with different GG concentrations directly after gel formation. Scale bars as indicated: 1 cm, 1 mm, and 40 µm. Data are representative of three independent experiments with three different biological donors.


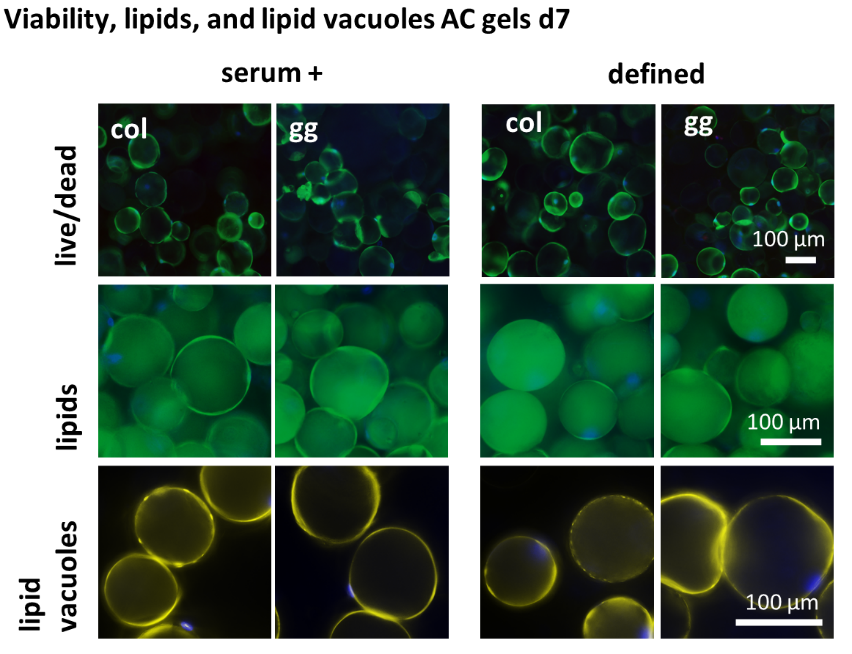


**Figure S4.** Comparison of collagen and gellan gum for encapsulation of mature adipocytes in serum-containing and defined medium on day 7. Live/dead staining of AC-col and AC-GG hydrogels in serum-containing medium and in the defined medium, living cells in green, dead cells in red, and nuclei in blue, scale bar 100 µm. Visualization of intracellular lipids and lipid vacuoles of encapsulated ACs in col and GG hydrogels at day 7. Lipids stained with BODIPY, and lipid vacuoles stained for perilipin A, lipids in green, lipid vacuoles in yellow, nuclei in blue. Scale bar 100 µm. Data are representative of three independent experiments with three different biological donors.


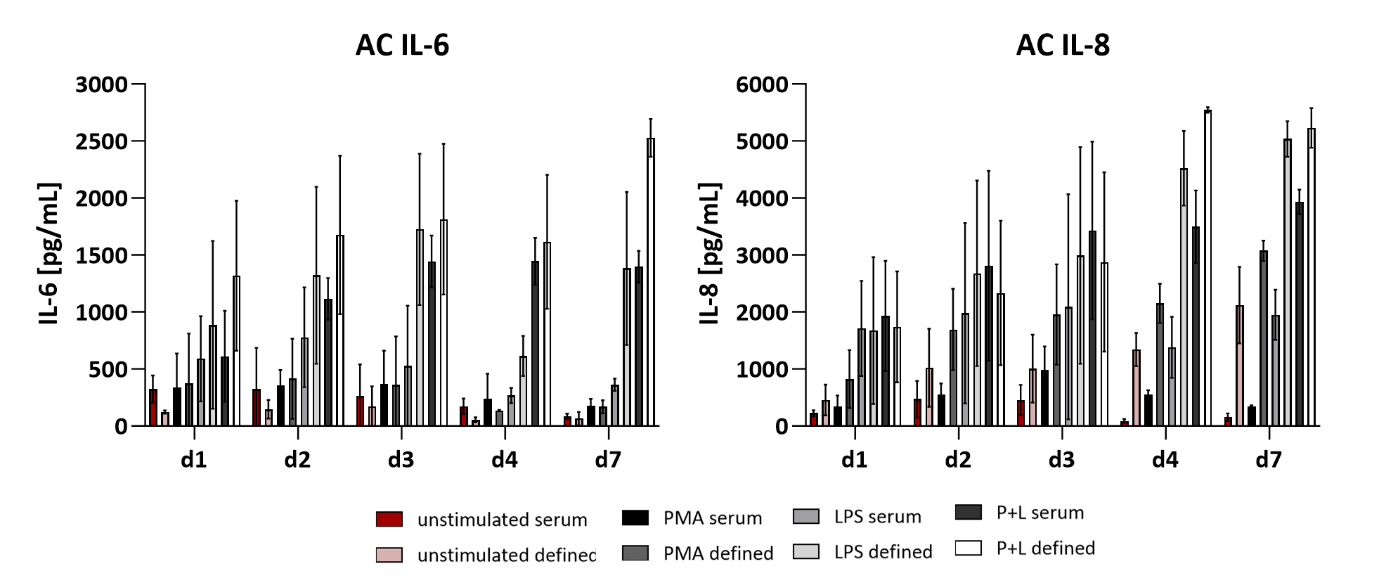


**Figure S5.** Release of cytokines IL-6 and IL-8 of unstimulated and PMA, LPS, or PMA+LPS-activated AC-GG gels in serum-containing and defined medium over 7 days. Mean values represent the mean ± SD in pg mL^-1^ of three independent experiments with three different biological donors.


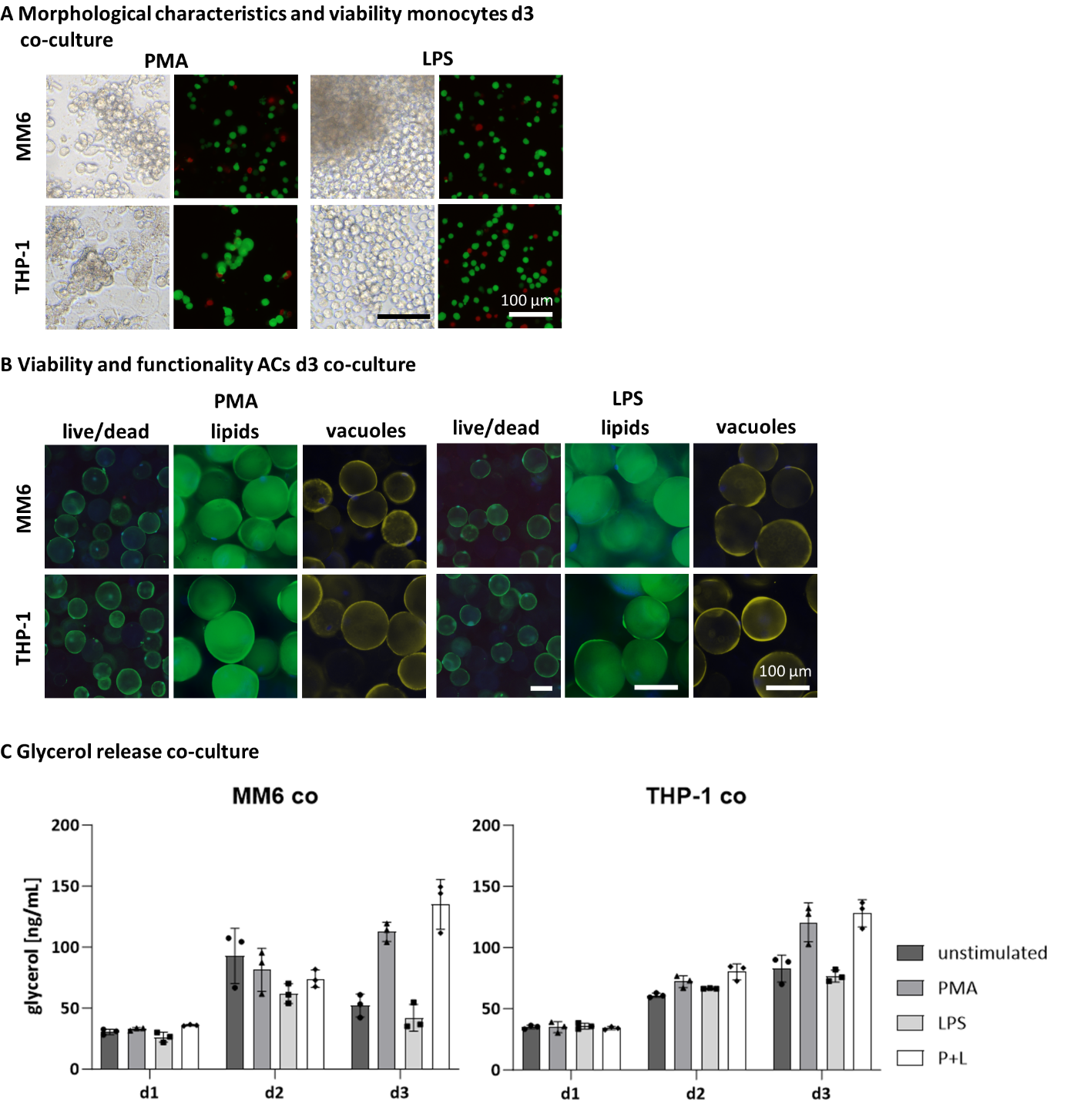


**Figure S6.** Viability, metabolic activity, and lipid content of the 3D adipocyte-macrophage co-culture after 3 days. A) Phase contrast images and live/dead staining of PMA or LPS-stimulated monocytes in co-culture on day 3, viable cells in green, dead cells in red. Scale bar 100 µm. B) Live/dead, intracellular lipid (BODIPY) and vacuole (perilipin A) staining of PMA or LPS stimulated co-cultured AC-GG hydrogels, viable cells in green, dead cells in red, lipids in green, perilipin A in yellow, nuclei in blue. Scale bar 100 µm. C) Basal glycerol release of unstimulated and stimulated co-culture in ng mL^-1^. Mean values represent the mean ± SD of three independent experiments with three different biological donors.


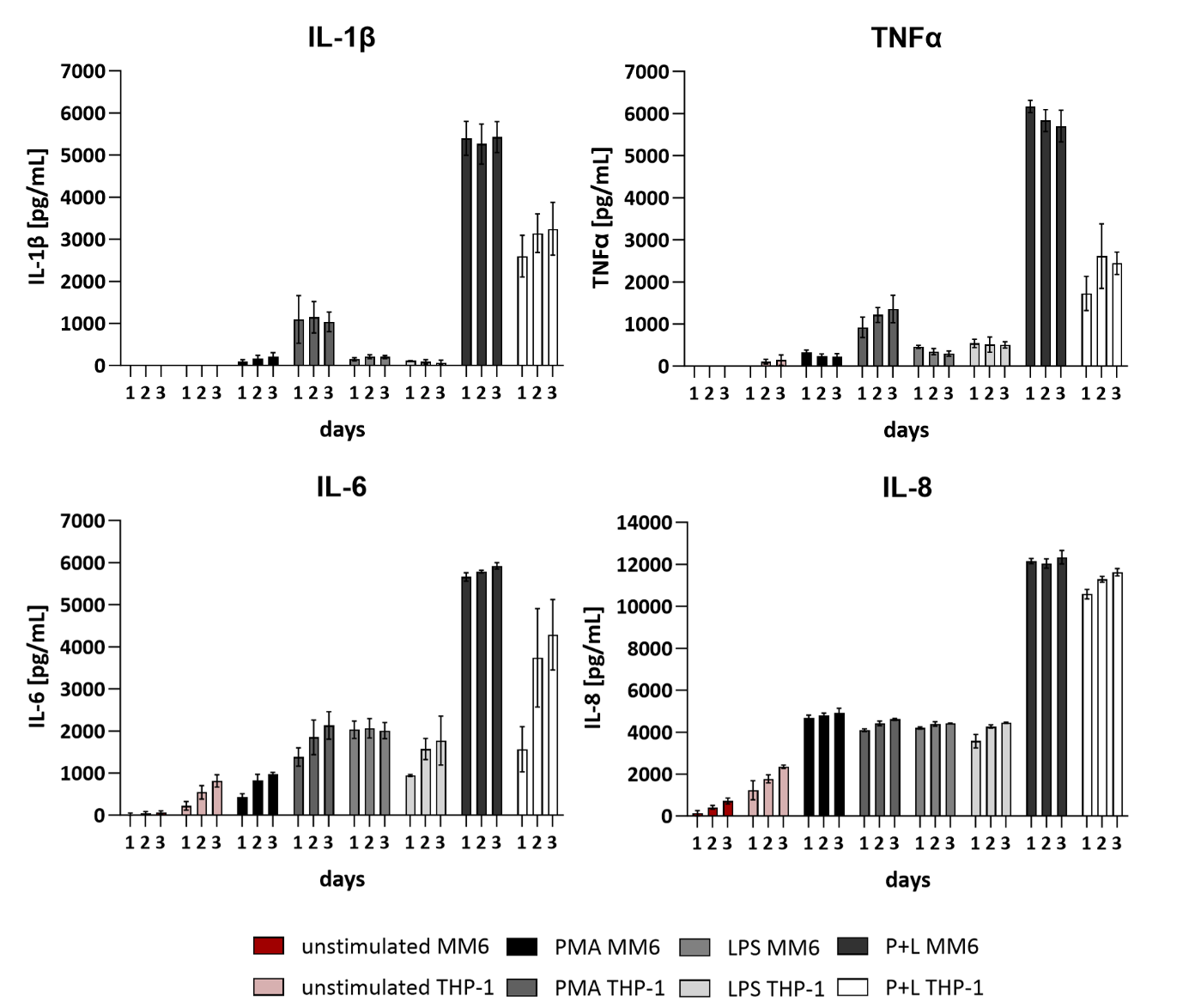


**Figure S7.** Cytokine release in co-culture over 3 days. IL-1β, TNFα, IL-6, and IL-8 in the supernatant of unstimulated and PMA, LPS, or PMA+LPS-activated co-culture (AC-MM6, AC-THP-1). Mean values represent the mean ± SD in pg mL^-1^ of three independent experiments with three different biological donors.

**Table S1.** Donor demographics of subcutaneous adipose tissue biopsies

| Experiment | Sex | Age | Source of biopsy | BMI at surgery |
| --- | --- | --- | --- | --- |
| Comparison col/GG | Female | 37 | Abdomen | 34.3 |
|  | Female | 48 | Thigh | 23.3 |
|  | Female | 55 | Thigh | 24.5 |
| AC monoculture activation | Female | 48 | Thigh | 23.3 |
|  | Female | 55 | Thigh | 24.5 |
|  | Female | 43 | Abdomen | 31.6 |
| AC co-culture activation | Female | 32 | Thigh | 28 |
|  | Female | 43 | Abdomen | 31.6 |
|  | Female | 46 | Abdomen | 30.5 |
